# Pan-transcriptomic Profiling Demarcates *Serendipita Indica*-Phosphorus Mediated Tolerance Mechanisms in Rice Exposed to Arsenic Toxicity

**DOI:** 10.1186/s12284-023-00645-0

**Published:** 2023-06-24

**Authors:** Shafaque Sehar, Muhammad Faheem Adil, Syed Muhammad Hassan Askri, Qidong Feng, Dongming Wei, Falak Sehar Sahito, Imran Haider Shamsi

**Affiliations:** 1 Zhejiang Key Laboratory of Crop Germplasm Resource, Department of Agronomy, College of Agriculture and Biotechnology, Zhejiang University, Hangzhou, 310058 China; 2 Dow International Medical College, Dow University of Health Sciences, Karachi, 74200 Pakistan

**Keywords:** Arsenic accumulation, Endophytic fungus, *Oryza sativa* L., Phytohormone, Transcriptome profiling

## Abstract

Inadvertent accumulation of arsenic (As) in rice (*Oryza sativa* L.) is a concern for people depending on it for their subsistence, as it verily causes epigenetic alterations across the genome as well as in specific cells. To ensure food safety, certain attempts have been made to nullify this highest health hazard encompassing physiological, chemical and biological methods. Albeit, the use of mycorrhizal association along with nutrient reinforcement strategy has not been explored yet. Mechanisms of response and resistance of two rice genotypes to As with or without phosphorus (P) nutrition and *Serendipita indica* (*S. indica; S.i*) colonization were explored by root transcriptome profiling in the present study. Results revealed that the resistant genotype had higher auxin content and root plasticity, which helped in keeping the As accumulation and P starvation response to a minimum under alone As stress. However, sufficient P supply and symbiotic relationship switched the energy resources towards plant’s developmental aspects rather than excessive root proliferation. Higher As accumulating genotype (GD-6) displayed upregulation of ethylene signaling/biosynthesis, root stunting and senescence related genes under As toxicity. Antioxidant defense system and cytokinin biosynthesis/signaling of both genotypes were strengthened under As + *S.i* + P, while the upregulation of potassium (K) and zinc (Zn) transporters depicted underlying cross-talk with iron (Fe) and P. Differential expression of phosphate transporters, peroxidases and GSTs, metal detoxification/transport proteins, as well as phytohormonal metabolism were responsible for As detoxification. Taken together, *S. indica* symbiosis fortified with adequate P-fertilizer can prove to be effective in minimizing As acquisition and accumulation in rice plants.

**Supplementary Information:**

The online version contains supplementary material available at 10.1186/s12284-023-00645-0.

## Background

Arsenic (As) becomes a constituent of food chain via its ingestion through contaminated water or food laden with As, which is either geogenically and/or anthropogenically released in the environment (Norton et al. 2012). Rice (*Oryza sativa* L.), due to its heavy irrigation requirement and cultivation in anaerobic soils, gets exposed to polluted water, making its grains tainted with inorganic arsenic species ‘As_*i*_’ (i.e., arsenate—As^V^ and arsenite— As^III^), and the organic forms dimethylarsinic acid, with trace amount of monomethylarsinic acid and tetramethylarsonium (Hansen et al. 2011). In China alone, almost 50% agricultural soil samples have verified As concentration of more than 40 mg kg^− 1^, which exceeds the threshold value as listed in “China Soil Environmental Quality (GB15618–2018)” (Lin et al. 2015). The related acute/chronic health damages for people on a rice-subsistence diet and the cumulative ravaging impacts on living cells, due to its competitive interference in P-reliant biological processes, make arsenic’s exclusion much more critical (Sehar et al. 2022). Because plant roots are the first organs coming in contact with xenobiotic compounds/stressors, and function at the interface, it is imminent to study the avoidance or tolerance mechanisms initiated at this end to cope with As stress. Having a strong inter-ionic competition between them (Boorboori & Zhang 2022), the chemical similarity of As^V^ with P leads to its uptake, rather indistinguishably, through phosphate transporters. In *Arabidopsis* root, disarray of the P transporters, e.g., *Pho1;1* and *Pho1;4*, provided tolerance to As^V^ (Shin et al. 2004). Kamiya et al. (2013) described PO_4_^3−^ deficiency signaling pathway as a target of As^V^ and the involvement of *OsPT1* in shoot As accumulation in rice. Moreover, knockout of *OsPT1* decreased As^V^ uptake and As_*i*_ accumulation in rice grains (Cao et al. 2017). As the P/As molar ratio is integral for the regulation of As mobility and accumulation, controlling the interactions between P and As in soil, however, is very complicated.

The results obtained from transcriptomic studies involving different cell types help us gain a deeper understanding regarding the constituents of a specific cell type, normal cellular functions, and the aberrations that may result from deviated gene activity (Huang et al. 2012). In order to understand the As toxicity mechanism, several microarray-based gene expression studies have been conducted (Dangleben et al. 2013). Arsenic exposure leads to adaptational changes, which encompass chromatin structure, transcriptome and the production of specific gene isoforms that, even upon withdrawal, do not lead to the complete recovery of chromatin structures and/or gene expression patterns (Riedmann et al. 2015). Norton et al. (2008) studied whole genome of rice roots exposed to arsenate in hydroponic conditions and denoted glutathione conjugation and As^V^ methylation as the most crucial biochemical responses to As^V^ stress. A separate analysis of As^V^ and As^III^ stresses at transcriptome level revealed differential expression of transporters, defense and stress-responsive genes, metallothioneins, sulfate-metabolizing and heat-shock proteins as well as regulatory genes, while the induction of cytochrome P450 genes occurred only under As^V^ stress (Chakrabarty et al. 2009). Furthermore, ethylene induced lignification occurred along with the upregulation of ATP-binding cassette superfamily transporters, ROS response network genes, mitogen-activated protein kinases (MAPKs) and calcium-dependent protein kinases (CDPKs), MYB and zinc-finger proteins, and senescence related (GARP-G2-like) transcription factors (Huang et al. 2012). Arsenate disrupts IAA biosynthesis, transport, and localization by negatively affecting the expression of IAA biosynthesis, i.e., *OsASA2* and *OsYUCCA2*, and transporter genes, i.e., *AUX1* and *PIN5b* (Ronzan et al. 2019). Arsenic notoriously impose iron (Fe) deficiency in plants, which in turn interferes with chlorophyll biosynthesis (Das and Sarkar 2018).

Comparatively, technology of bioremediation is taking precedence in attenuating As absorption in rice. According to prior reports, microbes could meliorate As accumulation by regulating As speciation, its bioavailability, and plant’s metabolism (Shri et al. 2019). Besides, the transcript levels of *OsLsi1* and *OsLsi2* were found to be repressed by arbuscular mycorrhizal fungi (AMF) in rice plants, thereby reducing As uptake/translocation (Shri et al. 2019). Transcriptional studies involving mycorrhizal fungus *Rhizophagus irregularis* colonization in rice roots resulted in a decrease in secondary cell-wall (SCW) related transcripts correlated with modified SCW composition; also, the hormonal profile and transport-related transcripts across different root types demonstrated a potential impact of symbiosis on root functioning and architecture (Gutjahr et al. 2015). In recent years, the focus of research has advanced to a specific group of AMFs, namely ‘Sebacinales’, including two phylogenetic-subgroups, i.e., Sebacinaceae and Serendipitaceae (Weiß et al. 2011); the latter contains several species, including *Serendipita indica* (formerly known as *Piriformospora indica*) found in Thar deserts of India (Varma et al. 1999), and others associated with plant family Orchidaceae (Oktalira et al. 2021). This endophyte is capable of establishing mutualistic association with many monocotyledonous and dicotyledonous plants (Franken 2012). Colonization of soybean roots with *S. indica* have been reported to instigate an upregulatory expression of abscisic acid-, gibberellin- and auxin- synthesis, signaling, or transport genes; where the auxin responsive genes displayed potential of reshaping root development and architecture (Bajaj et al. 2018). It has proven to adroitly tolerate, detoxify as well as immobilize As by decreasing its bioavailability via accumulation, adsorption and precipitation (Mohd et al. 2017; Ghorbani et al. 2021). Also, the remarkable P solubilization and utilization capabilities of this fungus assure its survival in the As-spiked growth conditions (Kushwaha et al. 2022). Pertinently, the employment of *S. indica* facilitates the uptake of available nutrients but cannot compensate the deficit thereof. Though several studies have delivered single puzzle pieces to the whole picture, the *S. indica* colonization-mediated arsenic tolerance in addition to P nutrition and related molecular mechanisms are quite understudied. In this work, the transcriptional changes of the two rice genotypes differing in their responses to As accumulation accompanied by P and *S. indica* colonization as nutrient management strategy and myco-remediative tool have been focused on, respectively.

## Materials and Methods

### Plant Material ***Serendipita Indica*** Culture Preparation and Inoculation

Healthy seeds of two rice genotypes, Zhongzhe You-1 (ZZY-1; G1; low As accumulator) and Guodao-6 (GD-6; G2; high As accumulator), were disinfected using 3% perhydroxic acid for about 15 min, then rinsed 3 times with ddH_2_O. Thereafter, the seeds of each genotype were soaked in a labeled germination box overnight at room temperature (25 °C). Afterward, the well moist seeds were placed for germination in a rice growth chamber at 29 °C ± 1 °C during the day (12 h) and 26 °C ± 1 °C at night. A hydroponic setup was arranged and 10 days old, uniform sized seedlings were transferred to half strength nutrient solution (Zeng et al. 2008), for 3 days followed by full strength solution for 4 days before fungal inoculation. Detailed composition of nutrient solution is mentioned in Additional file 1: Table S1. *Serendipita indica* was first grown on agar Petri plates with *Aspergillus* medium; circular agar disks (5 mm in diameter) inclosing active spores of *S. indica* (Hill and Käfer 2001), were placed on solidified medium and kept in absolute dark for 7 days at 30 °C ± 1 °C. For inoculation, *S. indica* was grown in a liquid Käfer medium from previously inoculated Petri plates; a small circular section of about 1 mm diameter of the inoculated fungus was introduced into a 500 mL glass flask and then incubated for 12 days at 30 °C ± 1 °C in a shaking incubator (200 rpm). Thereafter, the liquid containing active spores were filtered through sterile muslin cloth then used for plant inoculation by root soaking method (Chadha et al. 2015).

### Confirmation of ***Serendipita Indica*** Root Colonization and Treatment Application

Seven-day post-inoculation, successful colonization was confirmed by microscopic visualization of *S. indica* spores in the root cortical tissues of the host plant. Inoculated plant roots were first gathered and rinsed thoroughly with water before cutting them into 1 cm long pieces and then immersed in KOH solution (10%) overnight at room temperature (25 °C; Chadha et al. 2015). The roots were thereafter, washed three times with ddH_2_O and then immersed again, this time with 1% HCl for about 3 min prior to trypan blue (0.05%) staining. Followed by successful colonization, plants were kept either as positive control (*S.i*) or treated with alone 10 µM As L^− 1^ (As + *S. indica*), 50 µM P L^− 1^ (P + *S. indica*) and their combination (As + P + *S. indica*). A set of un-inoculated plants were also treated (As and As + P), meanwhile seedlings without any treatment were designated as control. With the aim of clarifying the transcriptomic profile of two genotypes, ZZY-1 (G1) and GD-6 (G2), under As (T1), As + P (T2), As + *S.i* (T3) and As + *S.i* + P (T4) treatments along with control (T5), cDNA from total 30 root samples (5 × 3 × 2, treatments×replicates×genotypes, respectively) was prepared and clean paired-end reads were subjected to sequencing. Arsenic (10 µM) was provided as Na_2_HAsO_4_.7H_2_O (0.0078 g L^− 1^), whereas for 50 µM phosphorus, 0.25 mM NaH_2_PO_4_.2H_2_O (0.039 g L^− 1^) was added (stock solutions).

### RNA Extraction, Library Preparation and Sequencing

Extraction of total RNA from the root tissues was carried out using TRIzol® reagent according to the instructions provided by manufacturer (Magen). Quantitative examination of RNA samples involved absorbance ratio analysis at A260/A280 through a Nanodrop ND-2000 system (Thermo Scientific, USA), and the integrity of RNA was determined by an Agilent Bioanalyzer 4150 system (Agilent Technologies, CA, USA). Paired-end library construction required ABclonal mRNA-seq Lib Prep Kit (ABclonal, China). Briefly, the purification of mRNA was done using l µg total RNA along with oligo (dT) magnetic beads, and subsequent fragmentation using divalent cations in ABclonal First Strand Synthesis Reaction Buffer at elevated temperatures. Accordingly, random hexamers were needed for the synthesis of first-strand cDNAs, while mRNA fragments served as templates for Reverse Transcriptase (RNase H), followed by synthesis of second-strand cDNA, which utilized DNA polymerase I, RNAseH, buffer and dNTPs. Post-synthesis, the double stranded cDNA fragments were adapter-ligated for paired-end library preparation, and later on used for PCR amplification. The resultant products were purified (AMPure XP system) and Agilent Bioanalyzer 4150 system assisted in assessing their quality. Libraries were sequenced using Illumina Novaseq 6000 (or MGISEQ-T7), which helped generate 150 bp paired-end reads. Afterwards, the bioinformatics analyses were performed using an in-house pipeline from Shanghai Applied Protein Technology Co., Ltd. The key softwares and procedures are as follows:

### Quality Control and Mapping

Firstly, the raw reads (in fastq format) were processed to obtain clean ones through perl scripts, which involved removal of the adapter sequence and filtering of low-quality reads (the number of lines with a string quality value ≤ 25 accounts for more than 60% of the entire reading), keeping N (undetermined base information) ratio > 5% of reads. The acquired reads were then aligned to reference genome separately, using HISAT2 software (http://daehwankim1ab.eithub.io/hisat2/), to obtain mapped reads.

### Quantitative Analysis of Differential Gene Expression Levels

Numbers of reads mapped to each gene were counted via FeatureCounts (http://subread.sourceforue.net/). In due course, the FPKM value for each gene was determined predicated on the length of the gene and corresponding mapped reads’ count. Analysis of differential expression was carried out using DESeq2 (http://bioconductor.ore/packages/release/bioc/html/DESeq2.html), where the DEGs with | log_2_FC | > 1 and *Padj* < 0.05 were designated as significantly different.

### Enrichment and Transcription Factor Analysis

Differential genes were evaluated and functionally annotated using Gene Ontology (GO) and Kyoto Encyclopedia of Genes and Genomes (KEGG) enrichment analyses to explain the differences between samples. To serve this purpose, clusterProfiler R software package was employed, where the values displaying *P* < 0.05 were considered significantly enriched for the GO or KEGG function in question. Correspondingly, TF analysis of DEGs involved direct data extraction from PlantTFDB database (http://planttfdb.cbi.pku.edu.cn/), whereas InterProScan tool (https://www.ebi.ac.uk/interpro/) assisted in annotating gene functions, and the predictive screening of putative transcription factors was carried out using DBD database (https://db1p.uni-trier.de/rec/journals/nar/Kummerfe1dT06.html).

### Verification of RNA-Seq Results with qRT-PCR

The RNA-Seq results were verified by performing qRT-PCR analysis following the methods discussed in detail by Adil et al. (2020). For internal reference, the rice actin gene (accession number XM_015774830) was used with the primer sequences: F— ^5^´TCCTCCGTGGAGAAGAGCTA^3^´ and R—^5^´GCAATGCCAGGGAACATAGT^3^´. Oligonucleotide primers for the nine genes related to sugar and metal transport, detoxification and cell-wall components were used, and the results determined by the 2^(−ΔΔCt)^ method (Schmittgen and Livak 2008), agreed with the whole transcriptome findings (displaying a correlation coefficient = 0.893) (Additional file 2: Fig. S1 and Additional file 1: Table S2).

### Statistical Analysis

Data were expressed as mean ± standard error of three biological replicates for each sample. Statistical analyses were performed using the Graphpad Prism (Graphpad Software, San Diego, CA, USA). Statistical significance was assessed at *P* < 0.05 significance level using Duncan’s multiple comparison.

## Results

With the aim of clarifying the transcriptomic profile of two genotypes, ZZY-1 (G1) and GD-6 (G2), under As (T1), As + P (T2), As + *S.i* (T3) and As + *S.i* + P (T4) treatments along with control (T5), cDNA from total 30 root samples was prepared and clean paired-end reads were subjected to sequencing. About 8.9 Gb reads sample^− 1^ were generated with a GC content of 46.9–51.66%, Q30 ˃ 92.8%, and an alignment efficiency of 69.5–90.1%, from which higher than 67% reads were uniquely mapped (Additional file 1: Table S3). Cumulatively, 59,833,545 clean reads were generated from each library. Most importantly, the sequencing quality was quite satisfactory to proceed analyzing further (Additional file 2: Fig. S2). Novel transcripts were merged with the reference sequence of *Oryza sativa* spp. *japonica*, thereafter clean reads were mapped and gene expression levels were calculated for each sample under control and four treatments exploiting RSEM tool (RNA-Seq by Expectation-Maximization). As shown in Additional file 1: Table S4, a total of 3860 (11.2%) novel genes were registered encoding proteins with unknown functions, including some that matched the description of putative CorA-like Mg^2+^ transporter protein [*Oryza sativa Japonica* Group] protein kinase domain (Novel.18,755), probable aquaporin TIP3-1 (Novel.30,957), NADH dehydrogenase subunit 6 (mitochondrion; Novel.10,674), mitochondrial outer membrane protein porin 2 (Novel.24,621), chloroplastic NADH-glutamate synthase 2 precursor (Novel.25,513).

### Gene Expression Analysis and Identification of DEGs

**Fig. 1:**
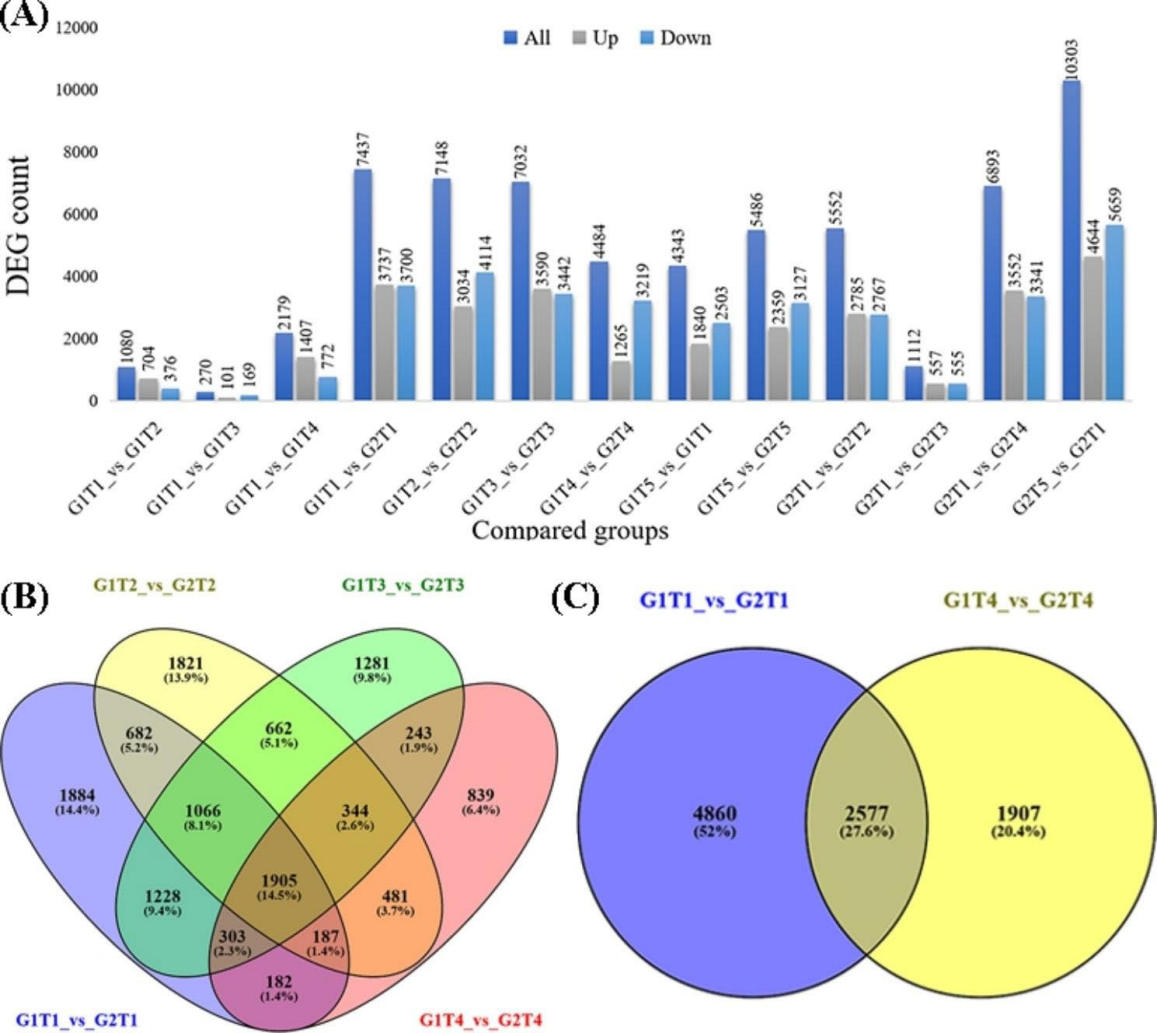
Comparative stats of differentially expressed transcripts and their counts (**A**) across the treatments between ZZY-1 and GD-6. Following treatments were employed: As_10µM_ (T1), As_10µM_+P_50µM_ (T2), As_10µM_+*S.i* (T3), As_10µM_+*S.i* + P_50 µM_ (T4) and control (T5). Venn diagram representing number of differentially expressed genes classified into differential interactions of As/*S.i*/P; (**B**) Overlaps among DEGs within group comparisons between ZZY-1 vs. GD-6 (G1T1 vs. G2T1, G1T2 vs. G2T2, G1T3 vs. G2T3 and G1T4 vs. G2T4); and (**C**) G1T1 vs. G2T1 and G1T4 vs. G2T4.

Out of 34,575 total genes, the number of differentially expressing genes was found highest in G2T5 vs. G2T1 (29.8%), followed by G1T1 vs. G2T1 (Fig. 1A). Pairwise comparisons displayed more downregulated genes than upregulated ones overall. Succinctly, the comparison, i.e., G1T1 vs. G1T2, G1T1 vs. G1T4, G1T1 vs. G2T1, G1T3 vs. G2T3, G2T1 vs. G2T2, G2T1 vs. G2T3 and G2T1 vs. G2T4, brought about a high number of upregulated DEGs, i.e., 65.2%, 64.5%, 50.2%, 51.1%, 50.2%, 50% and 51.5%, respectively. There were 2577 commonly expressed, while 4860 and 1907 uniquely expressed genes under As (G1T1 vs. G2T1) and As + *S.i* + P (G1T4 vs. G2T4), respectively, indicating that a huge number of genes expressed upon arsenic toxicity stimulus (Fig. 1B and C). Among the genotypes across the treatments, 839, 1281, 1821 and 1884 genes were uniquely expressed for G1T4 vs. G2T4, G1T3 vs. G2T3, G1T2 vs. G2T2 and G1T1 vs. G2T1, respectively, while 182 DEGs were expressed mutually.

Genotypic comparisons showed that ZZY-1 genotype had 353, 107, 828 and 2978 unique genes expressed differentially for G1T1 vs. G1T2, G1T1 vs. G1T3, G1T1 vs. G1T4 and G1T5 vs. G1T1, respectively, while 175 genes expressed mutually. Guodao-6 displayed 571, 156, 780 and 3464 DEGs when compared for G2T1 vs. G2T2, G2T1 vs. G2T3, G2T1 vs. G2T4 and G2T5 vs. G2T1, respectively, with 956 co-expressing genes (Additional file 2: Fig. S3). Collectively, the control group comparison of ZZY-1 and GD-6 (G1T5 vs. G2T5) presented 5486 DEGs, which setting against G1T1 vs. G2T1, G1T2 vs. G2T2, G1T3 vs. G2T3 and G1T4 vs. G2T4, shared 2854, 3190, 2730 and 2366 DEGs, respectively. On the one hand, when ZZY-1 control against As_10μM_ group (G1T5 vs. G1T1; 4343 DEGs) was compared with its own treatment groups, i.e., G1T1 vs. G1T2, G1T1 vs. G1T3 and G1T1 vs. G1T4, it shared 490, 107 and 1121 DEGs, respectively. On the other hand, GD-6 control against As_10μM_ group (G2T5 vs. G2T1; 10,303 DEGs) shared 4685, 818 and 5751 DEGs with G2T1 vs. G2T2, G2T1 vs. G2T3 and G2T1 vs. G2T4 groups, respectively.

**Fig. 2:**
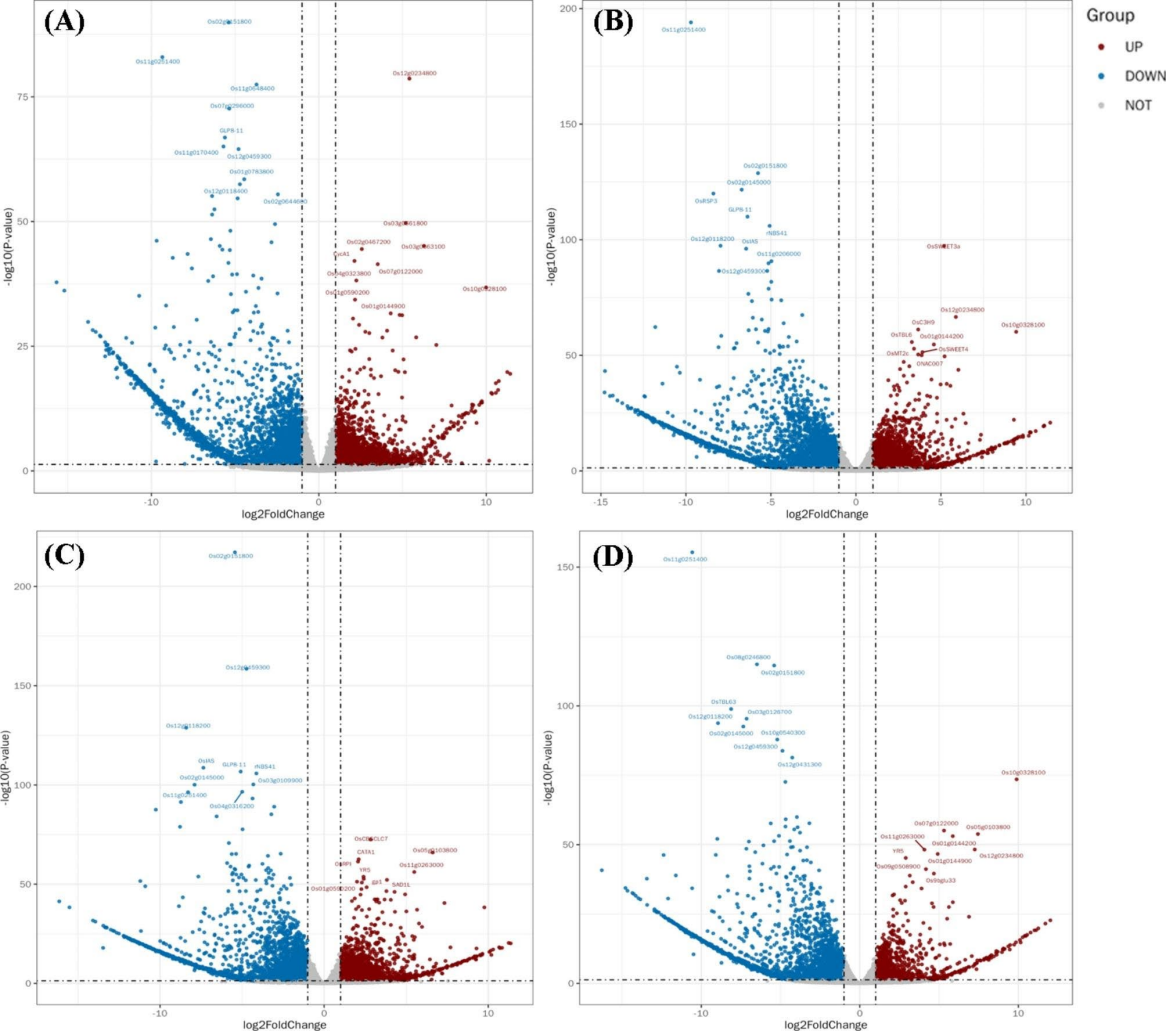
Volcanogram representing differentially expressed transcripts analyzed by DEseq2 in roots of two rice genotypes (G1—ZZY-1 and G2—GD-6) in response to As, P and *S. indica*. **(A)**, G1T1 vs. G2T1; **(B)**, G1T2 vs. G2T2; **(C)**, G1T3 vs. G2T3; and **(D**), G1T4 vs. G2T4. X axis represents log_2_ transformed fold change. Y axis represents -log_10_ (adjusted *P* value). T1 = As_10µM_, T2 = As_10µM_+P_50µM_, T3 = As_10µM_+*S.i* and T4 = As_10µM_+*S.i* + P_50 µM_. Maroon points represent upregulated DEGs, blue points represent downregulated ones and gray dots represent non-DEGs.

Correspondingly, principle component analysis (PCA) revealed that samples of the two genotypes were clearly separated by PC1 and PC2, which could explain for 64.7% of the total variation, signifying the differential genotypic behavior (Additional file 2: Fig. S4); moreover, the controls were clearly separated from treatments, indicating that arsenic toxicity posed a substantial impact on the transcript levels. Relevantly, the volcano plots for the pairwise treatment comparisons depicted that the highest number of down-regulated DEGs belonged to G1T2 vs. G2T2 (4114; 57.6%); whereas G1T4 vs. G2T4 reportedly exhibited the highest percentage (71.7%) of down-regulated genes (Fig. 2A-D). Among the genotype based pairwise comparisons, G1T1 vs. G1T3 presented 62.6% downregulated DEGs as opposed to the G1T1 vs. G1T2, G1T1 vs. G1T4 and G1T5 vs. G1T1 (34.8%, 35.4% and 57.6%, respectively). In case of GD-6, G2T5 vs. G2T1 comparison had higher percentage of down-regulated DEGs (54.9%) compared to its other three counterparts. Contingent on expression levels, all genes could be placed into 6 sub clusters (Additional file 2: Fig. S5) and 7 color modules (Additional file 2: Fig. S6).

### Pathway Analyses and Functional Enrichment of DEGs via KEGG

**Fig. 3:**
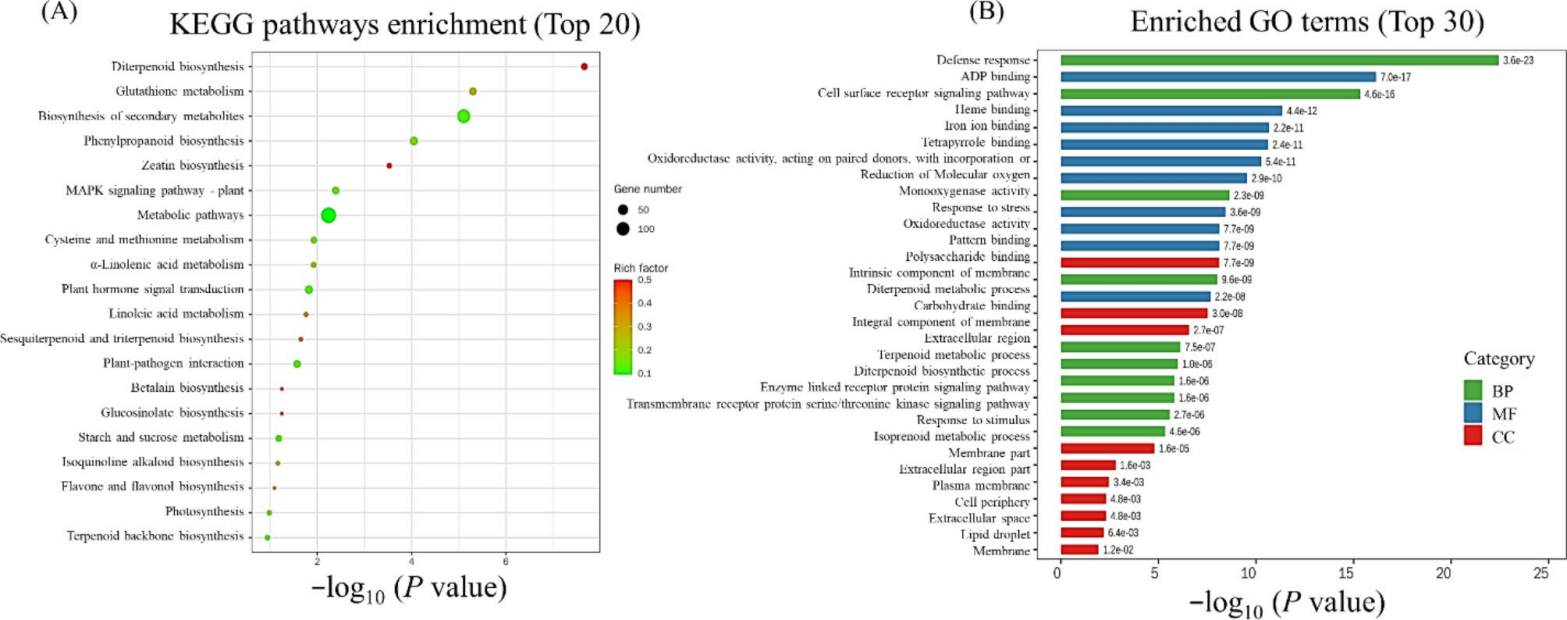
KEGG analysis of DEGs presented as scatter plot showing 20 most significantly enriched pathways **(A)**, and relative comparative gene ontology (GO) in ZZY-1 under As_10__µM_ + *S.i* + P_50µM_ in comparison to GD-6 **(B)**. Each circle in the dot plot represents a pathway and the size of the circle represents the number of genes enriched in the pathway. The ordinate represents the pathway name and the abscissa represents the rich factor, which compares the ratio of genes annotated to a pathway among the DEGs to the ratio of genes annotated to that pathway among all genes. The color of the circle represents the *q*-value, where smaller values equate to more reliable enrichment significances of the DEGs in the pathway. GO terms were sorted based on *q*-values (< 0.05) and belonged to biological processes, cellular components, and molecular functions were shown in green, red and blue color, respectively

Information related to the expressed genes and their putative functions were determined through KEGG pathway classification/functional enrichment analysis (Fig. 3A and S7). During sorting, the smallest *P*-values (< 0.05) were given weightage and the number of genes enriched in the first 20 entries were counted. Among the top 20 KEGG pathways, ‘linoleic acid metabolism’, ‘sesquiterpenoid and triterpenoid biosynthesis’ and ‘phenylpropanoid biosynthesis’ were enriched mutually for all treatment wise comparisons. ‘Starch/ sucrose metabolism’ and ‘α-linolenic acid metabolism’ were enriched mutually for G1T1 vs. G2T1, G1T3 vs. G2T3 and G1T4 vs. G2T4, while ‘flavone and flavonol biosynthesis’, plant hormone signal transduction’ ‘MAPK signaling’, ‘biosynthesis of secondary metabolites’, ‘diterpenoid biosynthesis’, ‘isoquinoline alkaloid biosynthesis’ and ‘plant-pathogen interaction’ were inclusively and significantly enriched KEGG pathways for G1T2 vs. G2T2, G1T3 vs. G2T3 and G1T4 vs. G2T4 comparisons. G1T1 vs. G2T1 and G1T2 vs. G2T2 (Additional file 2: Fig. S7) had common and significant enrichment of ‘ribosome’ and ‘pyruvate metabolism’; G1T3 vs. G2T3 and G1T4 vs. G2T4 were enriched for ‘metabolic pathways’ and ‘cysteine and methionine metabolism’; for the comparisons of G1T2 vs. G2T2 and G1T4 vs. G2T4, it was related to ‘glutathione metabolism’ and ‘photosynthesis’; G1T2 vs. G2T2 and G1T3 vs. G2T3 displayed significant enrichment of ‘cutin, suberin and wax biosynthesis’; additionally, G1T1 vs. G2T1 and G1T4 vs. G2T4 shared ‘terpenoid backbone biosynthesis’, whereas for G1T1 vs. G2T1 and G1T3 vs. G2T3, ‘fatty acid biosynthesis’, ‘fatty acid metabolism’, ‘DNA replication’ and ‘biotin metabolism’ were expressed as commonly enriched pathways. In particular, the G1 vs. G2 arsenic stress (T1) group showed ‘purine metabolism’, ‘fatty acid degradation’, ‘phagosome’, ‘cyanoamino acid metabolism’, ‘benzoxazinoid biosynthesis’, ‘steroid biosynthesis’, ‘valine, leucine and isoleucine degradation’ and ‘glycosphingolipid biosynthesis - lacto and neolacto series’, to be significantly enriched. Furthermore, ‘flavonoid biosynthesis’, ‘pentose and glucuronate interconversions’, ‘carotenoid biosynthesis’, ‘tropane, piperidine and pyridine alkaloid biosynthesis’, and ‘tyrosine metabolism’ enriched for G1T2 vs. G2T2; whereas, the uniquely enriched pathway for G1T3 vs. G2T3 was ‘nitrogen metabolism’. ‘Zeatin biosynthesis’, ‘betalain biosynthesis’, and ‘glucosinolate biosynthesis’ were exclusively enriched for G1T4 vs. G2T4 (Fig. 3A and Additional file 2: Fig. S7).

### Gene Ontological Classification of DEGs

In pursuit of functionally annotating the DEGs further, gene ontological categories were investigated including the domains: biological processes (BPs), cellular components (CCs), and molecular functions (MFs) (Fig. 3B and Additional file 2: Fig. S8). The enrichment results implied that ‘ADP binding’ was commonly enriched across the treatment wise paired comparisons among the molecular functions; however, ‘structural constituent of ribosome’, ‘structural molecule activity’, ‘rRNA binding’, ‘large ribosomal subunit rRNA binding’, ‘nutrient reservoir activity’, ‘O-acetyltransferase activity’, ‘small ribosomal subunit rRNA binding’, ‘D-threo-aldose 1-dehydrogenase activity’, ‘oxidoreductase activity, acting on the CH-OH group of donors, NAD or NADP as acceptor’, were significantly and exclusively enriched for G1T1 vs. G2T1. The enrichment of gene ontological terms, i.e., ‘heme binding’, ‘tetrapyrrole binding’, ‘oxidoreductase activity’, ‘oxidoreductase activity, acting on paired donors, with incorporation or reduction of molecular oxygen’ and ‘monooxygenase activity’, were found to be mutual for G1T2 vs. G2T2, G1T3 vs. G2T3 and G1T4 vs. G2T4. The GO terms: ‘oxidoreductase activity, acting on peroxide as acceptor’ and ‘peroxidase activity’ were highlighted for G1T2 vs. G2T2 and G1T3 vs. G2T3, inclusively. While, G1T2 vs. G2T2 had an exclusive enrichment of ‘hydroquinone: oxygen oxidoreductase activity’ and ‘glucosyltransferase activity’; G1T3 vs. G2T3 had ‘microtubule binding’, ‘tubulin binding’; and G1T4 vs. G2T4 had ‘iron ion binding’, ‘pattern binding’, ‘polysaccharide binding’, as well as ‘carbohydrate binding’.

The G1T1 vs. G2T1 did not share any term in relation to cellular components or biological functions among the top 10 enriched GO terms with the other three comparisons, whereas ‘cell periphery’, ‘extracellular region’, ‘intrinsic component of membrane’, ‘integral component of membrane’ and ‘plasma membrane’ were the commonly CCs enriched for G1T2 vs. G2T2, G1T3 vs. G2T3, G1T4 vs. G2T4. For G1T2 vs. G2T2 and G1T3 vs. G2T3 comparisons, the shared terms were ‘apoplast’, ‘external encapsulating structure’, ‘cell-wall’, ‘anchored component of membrane’ and ‘anchored component of plasma membrane’. Exclusively, ‘membrane part’, ‘extracellular region part’, ‘extracellular space’, ‘lipid droplet’, and ‘membrane’ were enriched for G1T4 vs. G2T4. Among the most enriched and mutually expressed biological process term for G1T2 vs. G2T2, G1T3 vs. G2T3 and G1T4 vs. G2T4, was ‘defense response’. Additionally, genes governing ‘response to stress’ were commonly highlighted for G1T2 vs. G2T2 and G1T4 vs. G2T4. In case of G1T2 vs. G2T2 and G1T3 vs. G2T3, ‘cell-wall organization or biogenesis’ expressed mutually. Interestingly, the BPs up-regulated for the G1T4 vs. G2T4 involved ‘telomere maintenance via telomerase’, ‘telomere maintenance via telomere lengthening’, ‘inflorescence development’, ‘reproductive shoot system development’, ‘regulation of salicylic acid biosynthetic process’, ‘salicylic acid biosynthetic process’, ‘regulation of salicylic acid metabolic process’, along with ‘RNA-dependent DNA biosynthetic process’, ‘antibiotic biosynthetic process’ and ‘response to hydrogen peroxide’ terms, which are unique terms for this treatment-wise comparison.

Other groups displayed biological processes: ‘cell cycle’, ‘mitotic cell cycle’, ‘mitotic cell cycle process’, ‘cell cycle process’, ‘microtubule-based process’, ‘cell division’ exclusive for G1T3 vs. G2T3; ‘lignin catabolic process’, ‘phenylpropanoid catabolic process’, ‘cell-wall biogenesis’, ‘plant type cell-wall biogenesis’, ‘plant-type secondary cell-wall biogenesis’ as well as ‘external encapsulating structure organization’ for G1T2 vs. G2T2; however for G1T1 vs. G2T1, most BPs were unique and associated with ‘peptide biosynthetic process’, ‘amide biosynthetic process’, ‘peptide metabolic process’, ‘organonitrogen compound biosynthetic process’, ‘cellular nitrogen compound biosynthetic process’, ‘ribosome assembly’, ‘cellular amide metabolic process’, ‘cellular macromolecule biosynthetic process’ and ‘macromolecule biosynthetic process’ (Additional file 2: Fig. S8).

### Transcription Factors

**Fig. 4:**
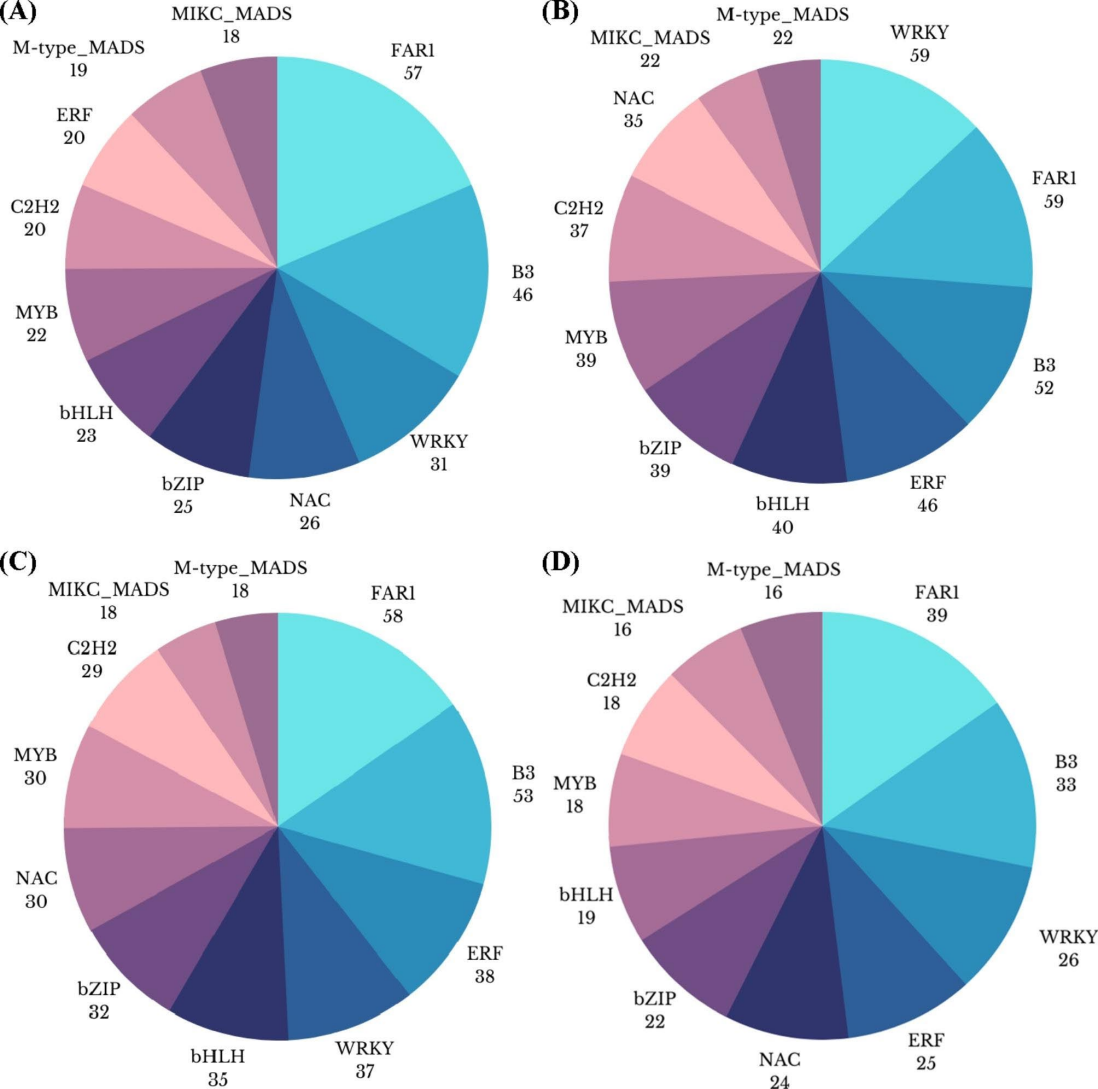
Comparative stats of differentially expressed transcription factors across the treatments in two rice genotypes differing in their arsenic tolerance response; (**A**) G1T1 vs. G2T1, (**B**) G1T2 vs. G2T2, (**C**) G1T3 vs. G2T3, and (**D**) G1T4 vs. G2T4.

A number of transcription families were exclusively/ inclusively expressed, including WRKY, FAR1, NAC, bZIP, bHLH, B3, C2H2, ERF, M-type-MADS, MIKC-MADS, MYB- and -related. It is worth pointing out that a number of genes related to FAR1 TF (57) were identified for G1T1 vs. G2T1 (out of 452 genes), while out of 598 genes associated with TFs under G1T2 vs. G2T2, WRKY and FAR1 were on a par (59 genes each). Moreover, from 548 DEGs, 58 were identified belonging to FAR1 followed by B3 TF with 53 genes for G1T3 vs. G2T3. A comparatively smaller number of TF related gene expression was observed (Fig. 4) for G1T4 vs. G2T4 as out of 332 DEGs, most were associated with FAR1 (39) and B3 (33).

### Heat Shock and Stress Responsive DEGs

**Fig. 5:**
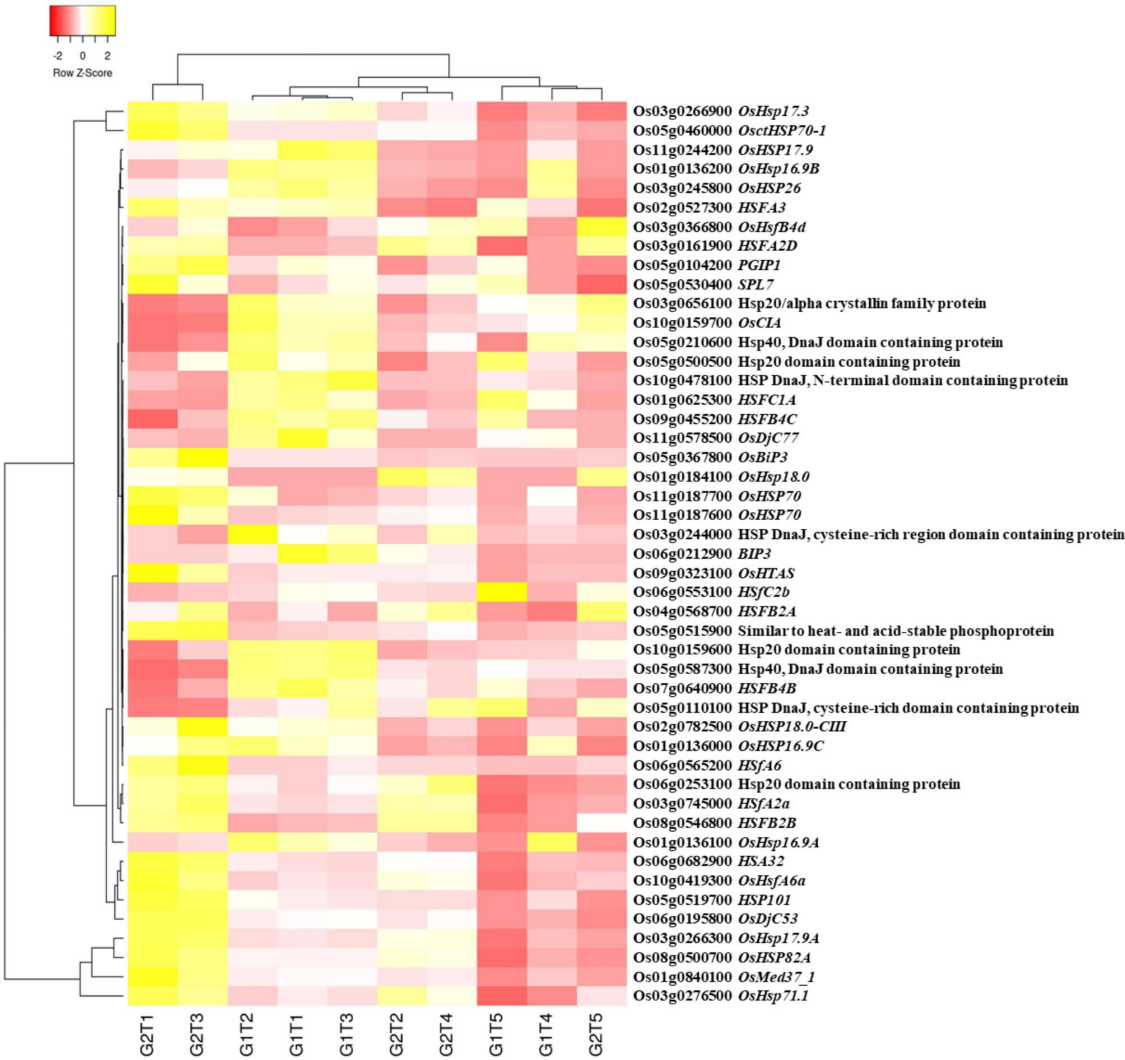
Hierarchical heat map showing the expression profile of inclusively expressed heat shock proteins and TFs in response to As alone (T1), As + P (T2), As + *S.i* (T3) and As + *S.i* + P (T4) treatments along with control (T5), in the two rice genotypes ZZY-1 (G1) and GD-6 (G2)

A total of 215 stress-related DEGs were found being inclusively and exclusively expressed, including heat shock proteins and heat stress TFs, LEA proteins, zinc finger stress associated proteins, drought-, salt- and cold-tolerance related genes, PIPs and others. Regulation of stress related DEGs occurred in a treatment and genotype dependent manner (Additional file 2: Fig. S9). Heat shock protein 17.9 kDa (Os11g0244200) and *OsHsp16.9B* (Os01g0136200) were not expressed in the controls of both genotypes but the expression was observed across all treatments, more so in ZZY1; whereas, a heat shock protein 70 (Os11g0703900) did not express under the control (T5) and As_10__µM_ + *S.i* + P_50µM_ treatment (T4) for both the genotypes. A remarkable increase in the expressions of *OsHSP82A* (Os08g0500700), *OsHsp71.1* (Os03g0276500), *OsHsp17.3* (Os03g0266900), *OsctHSP70-1* (Os05g0460000), and *OsMed37_1* (Os01g0840100) was witnessed across all treatments in both genotypes against their respective controls (Fig. 5). Exclusively under GD-6, *OsHsp18.0* (Os01g0184100) was expressed, moreover a protein involved in negative salt stress tolerance (*OCPI2*; Os01g0615100) had higher expression under arsenic treatment (T1) in GD-6 than ZZY-1. Stress response related lipid transport protein *OsACBP1* (Os08g0162800) expressions were found much higher in ZZY-1 genotype compared to GD-6 under all treatments. Meanwhile, the expression of *OsCCD1* (Os06g0683400) was higher in controls of both genotypes, albeit the exposure of arsenic undoubtedly reduced the levels regardless of the amelioratory variables.

### Detoxification Related DEGs

**Fig. 6:**
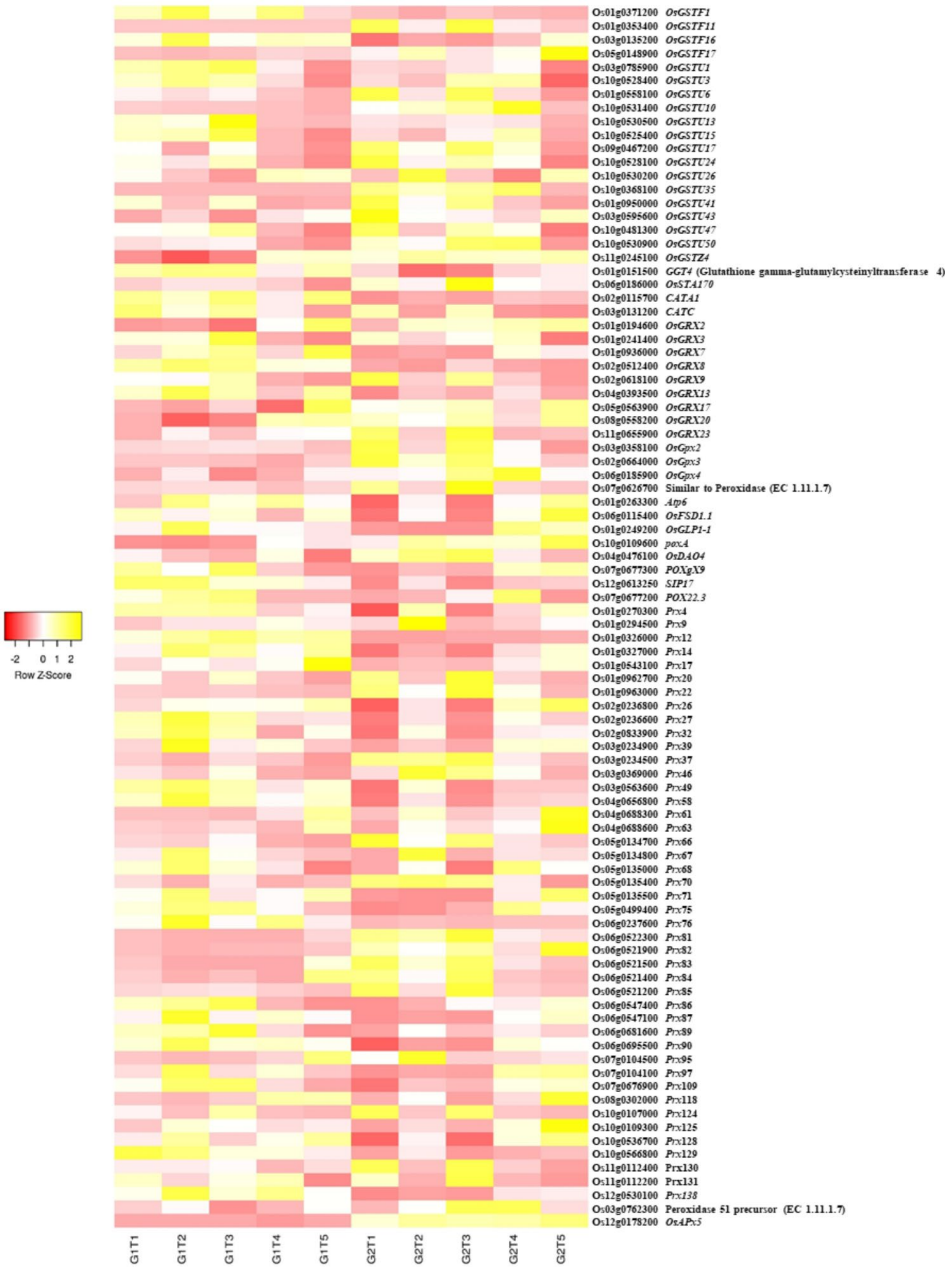
Heat map depicting the expression profile of inclusively expressed detoxification related DEGs in response to As alone (T1), As + P (T2), As + *S.i* (T3) and As + *S.i* + P (T4) treatments along with control (T5) in the two rice genotypes ZZY-1 (G1) and GD-6 (G2)

Inclusively and exclusively, around 167 genes were identified to be expressed differentially under this category. It was observed that a higher number of detoxification related genes were expressed under *S.i* colonization (T3) as compared to other treatments, reaching more than 6 log_2_-FC, with higher percentage of upregulated expression levels. Numerous peroxidases (POD), glutathione S-transferases, glutathione synthases, glutaredoxins were regulated by the treatments applied. The log_2_-FPKM values showed higher expression levels of *Os1-CysPrxA* (Os07g0638300) in ZZY-1 than GD-6 across all treatments against controls, which plainly depicts the genotypic difference in response to As exposure. Compared to ZZY-1, GD-6 displayed higher expression of *OsAPx5* (Os12g0178200). Furthermore, *OsGSTU35* (Os10g0368100) and *OsGSTU50* (Os10g0530900) were also expressed highly under all treatments for GD-6 in comparison with its own control and ZZY-1 (Fig. 6 and Additional file 1: Table S5).

### Transporters and Heavy Metal Related DEGs

**Fig. 7:**
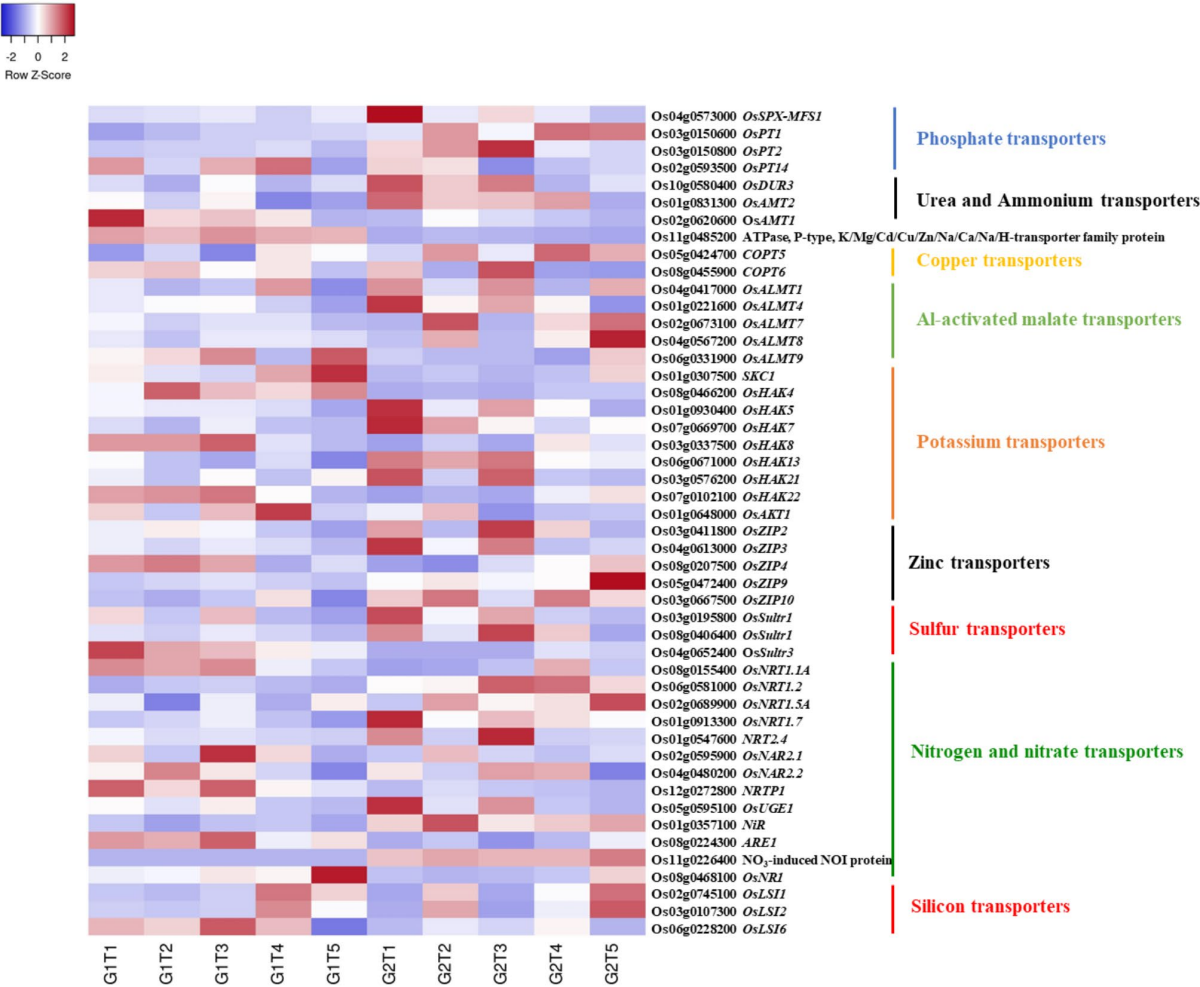
Heat map displaying the expression profile of inclusively expressed nutrient transporters in response to arsenic alone (T1), As + P (T2), As + *S.i* (T3) and As + *S.i* + P (T4) treatments along with control (T5), in the two rice genotypes ZZY-1 (G1) and GD-6 (G2)

A number of transport related DEGs (52) were identified in this study, including ABC (ATP-binding cassette) transporter related DEGs that belonged to Multidrug Resistance (MDR) and members of A, B, C, E, G and I families, phosphate (12), Zn (6), potassium (12), sulfate (9), copper (2), ammonium (2), nitrate (22), silicon (2), aluminium-induced malate transporters (7) and heavy metal related genes (112). Differences were observed in the expression of the genes among treatments and between genotypes (Fig. 7 and Additional file 1: Table S6). The expression of ABC transporters varied under different treatments for both genotypes; for genotype GD-6, these transporters had elevated expression levels under alone As_10_ stress than in combined treatments. However, *OsABCG16* (Os06g0731200) and *OsABCG21* (Os09g0401100) expressed highly under T4, *OsABCG13* (Os05g0384600), *OsABCG5* (Os03g0281900), *OsABCG3* (Os01g0836600) under T3, whereas *OsABCG2* (Os01g0615500) and *OsABCB25* (Os03g0755900) under T2 (Additional file 2: Fig. S10 and Fig. S11).

Upon As addition, the expression of *OsSPX-MFS1* (Os04g0573000) was increased comparatively more under As alone treatment in GD-6 than ZZY-1. Another phosphate transporter *OsPT1* (Os03g0150600) was altered noticeably, experiencing downregulation for ZZY-1 under all treatments against its control, albeit an increased expression was observed under all treatments including control in GD-6. The transcript levels of *OsPT14*, which function in Pi transport in subcellular organelles, increased under T1, T3 and T4 treatments for ZZY-1 but in case of GD-6, this increase was observed mainly under T1 and T2. Ammonium transporter 1 (Os02g0620600) seemed to have a higher expression in ZZY-1 under T1 in comparison with its T2, T3 and T4 counterparts as well as GD-6 genotype. However, *OsAMT2* (Os01g0831300) and *OsDUR3* (Os10g0580400) expressions were positively highlighted under As stress for GD-6. Copper transporter 5 (Os05g042470) displayed expression under T2 and T4 for both genotypes, more prominently in GD-6, while *OsCOPT6* (Os08g0455900) had higher expressions under T1 and T3 for GD-6, and T1 and T2 for ZZY-1. Aluminum-activated malate transporters were also found to express differentially, exhibiting high expressions generally in GD-6. Interestingly, a couple of differentially expressed genes associated with potassium transport were registered, with an elevated expression for both genotypes but under different treatments; *SKC1* (Os01g0307500) and *OsHAK4* (Os08g0466200) portrayed a higher expression in the controls of ZZY-1 than GD-6. Although, *OsHAK5* (Os01g0930400), *OsHAK7* (Os07g0669700), *OsHAK13* (Os06g0671000) and *OsHAK21* (Os03g0576200) were expressed more under T1-T3 for GD-6, *OsHAK8* (Os03g0337500) and *OsHAK22* (Os07g0102100) expressed for ZZY-1 under the same three treatments. Furthermore, *OsAKT1* (Os01g0648000) was expressed the most under T4 for ZZY-1. Among the NO_3_^–^ transporters, similar to nitrate-induced NOI protein (Os11g0226400), expressed only for GD-6, whereas silicon transporters *LSI1* (Os02g0745100) and *LSI2* (Os03g0107300), being highly regulated under control conditions, mostly downregulated under T1 and T3, though T4 in case of both genotypes and T2 in case of GD-6 only brought about higher expressions of these transporters. On the contrary, *LSI6* (Os06g0228200) significantly upregulated under all treatments for both genotypes, more profoundly for ZZY-1 (Additional file 1: Table S6).

### DEGs Associated with Phytohormones

**Fig. 8:**
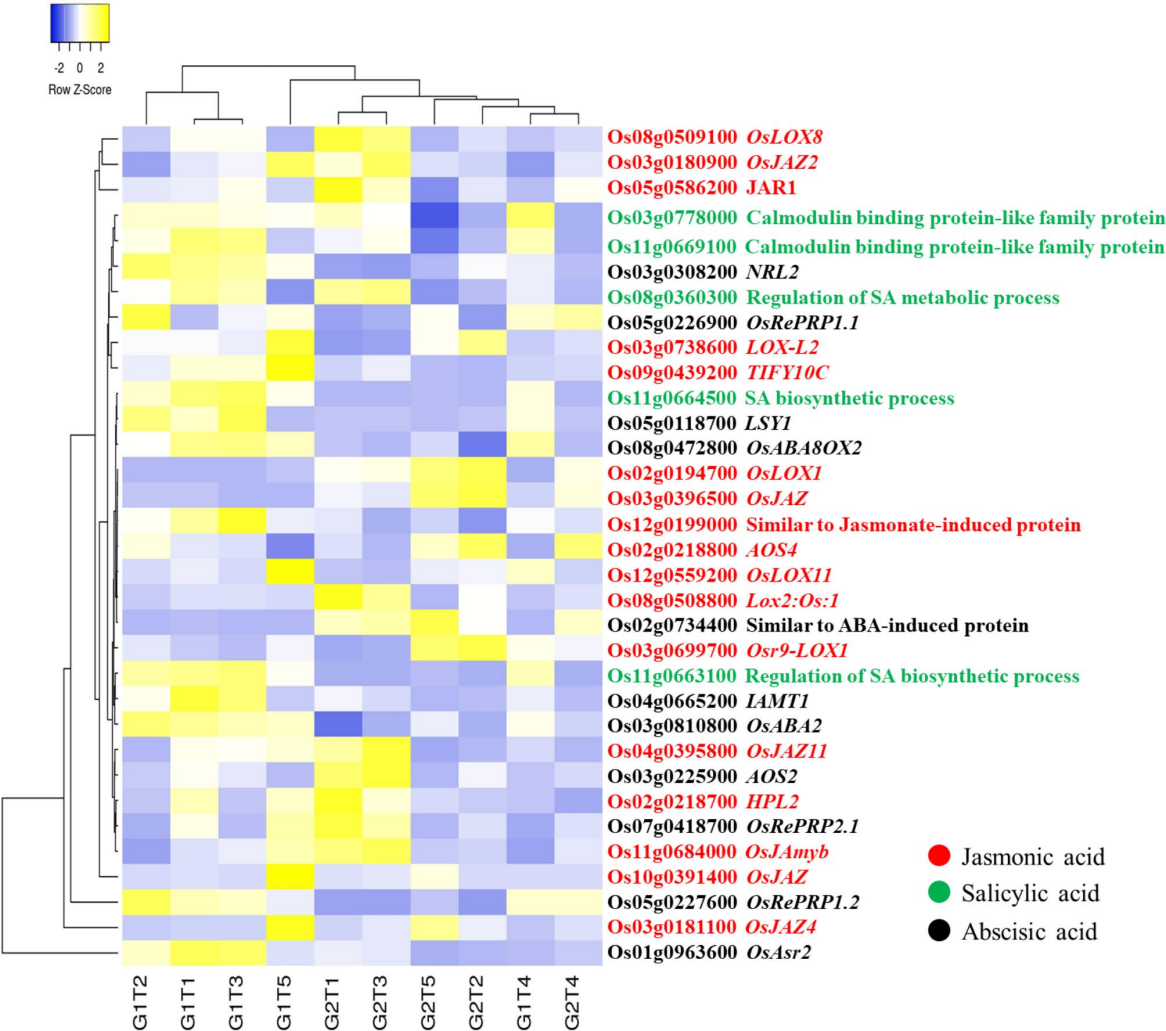
Hierarchical heat map showing the expression profile of inclusively expressed genes related to the biosynthesis and signaling of JA, SA and ABA in response to As alone (T1), As + P (T2), As + *S.i* (T3) and As + *S.i* + P (T4) treatments along with control (T5), in the two rice genotypes ZZY-1 (G1) and GD-6 (G2)

**Fig. 9:**
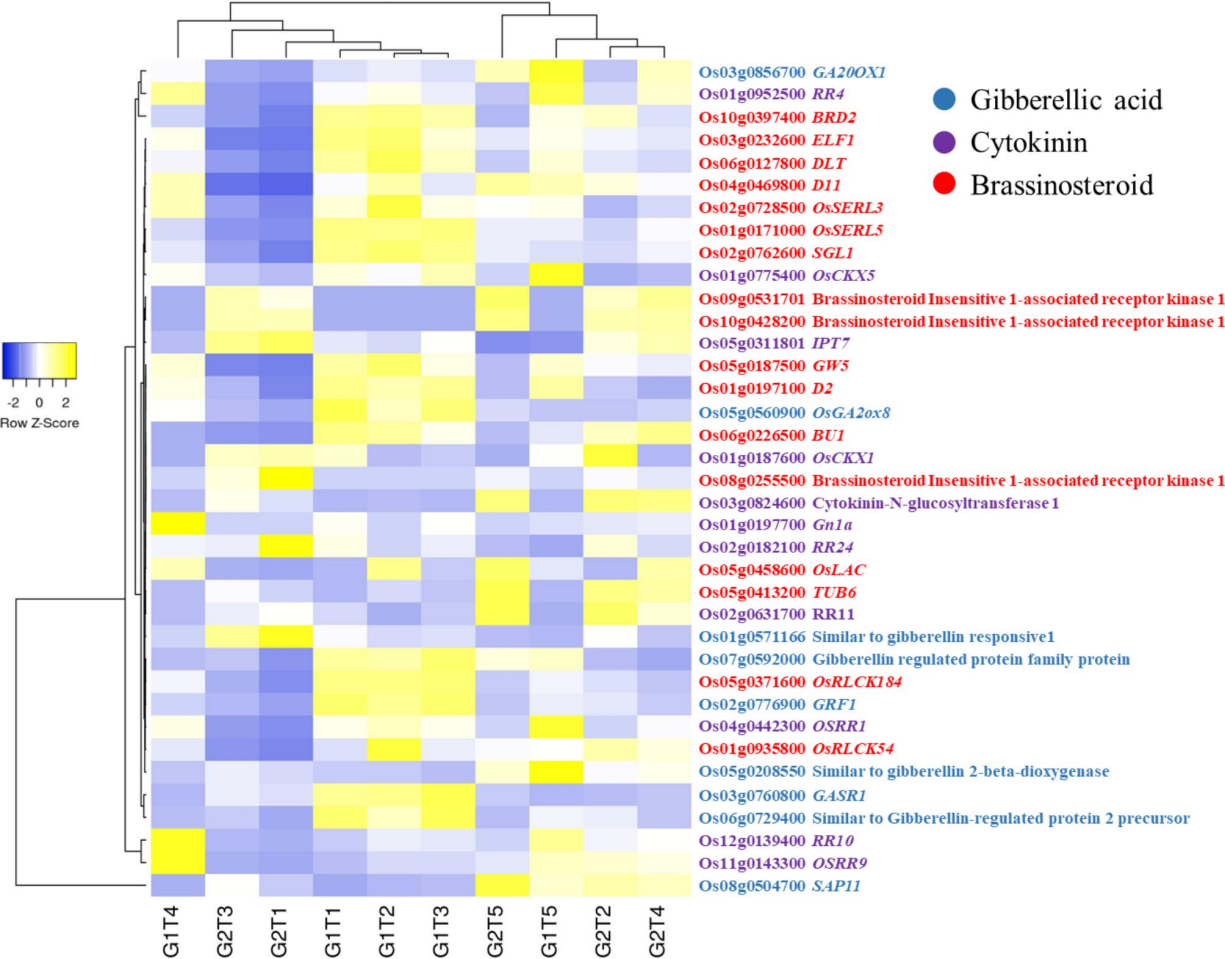
Hierarchical heat map illustrating the expression profile of inclusively expressed genes related to gibberellin, cytokinin and brassinosteroid biosynthesis and signaling in response to As alone (T1), As + P (T2), As + *S.i* (T3) and As + *S.i* + P (T4) treatments along with control (T5), in the two rice genotypes ZZY-1 (G1) and GD-6 (G2)

Owing to their crucial roles in abiotic stress regulation, a mutual comparison of DEGs related to auxin (Additional file 2: Fig. S12), ethylene (Additional file 2: Fig. S13), abscisic acid, jasmonic acid, salicylic acid (Fig. 8), cytokinin and gibberellic acid (Fig. 9), was performed. There were 255 inclusively and exclusively expressed DEGs, and a very large number of auxin related genes were upregulated for ZZY-1 regardless of the treatment applied, mostly being under T2, but DEGs such as *OsSAUR37* (Os09g0437100), similar to *SAR1* (suppressor of auxin resistance1; Os02g0142950) and *OsSAUR57* (Os12g0609600), were prominently expressed under T4 for GD-6 genotype. Moreover, similar to auxin-binding protein, *OsABP20* (Os04g0288100) expressed exclusively under GD-6. In terms of DEGs involved in ethylene biosynthesis or signaling, GD-6 exhibited innately higher expression as compared to ZZY-1, though an increase in expression was found under T1 and T3 for ZZY-1 as well, mostly under T2 and T3. However, *ACO4* (Os11g0186900) and *ACO5* (Os05g0149400) were highly expressed for this genotype under T4. Also, similar to ethylene-responsive transcriptional coactivator (Os06g0592500) gene was dramatically increased in its expression with the onset of As added treatments, where T1 and T3 enforced higher expressions in GD-6 compared to its T2 and T4 counterparts but also against ZZY-1. The expressions of brassinosteroids associated DEGs were comparatively higher in ZZY-1, specially under T1, T2 and T3; moreover, brassinosteroid insensitive 1-associated receptor kinase 1 (Os08g0255500), expressed exclusively for GD-6. Differentially expressed genes involved in gibberellin regulation were significantly expressed for ZZY-1 genotype, while GD-6 had a fair share of upregulated cytokinin related DEGs, quite noticeable under T2, T3 and T4. A relatively higher number of ABA and SA related DEGs were expressed significantly and positively for ZZY-1 compared to GD-6 genotype, which presented an affinity towards jasmonic acid associated DEGs’ upregulation under its As stressed treatments as opposed to ZZY-1. The comparisons revealed that G1T3 vs. G2T3 had a very strong auxin signaling response (Figs. 8 and 9, Additional file 1: Table S7).

**Fig. 10:**
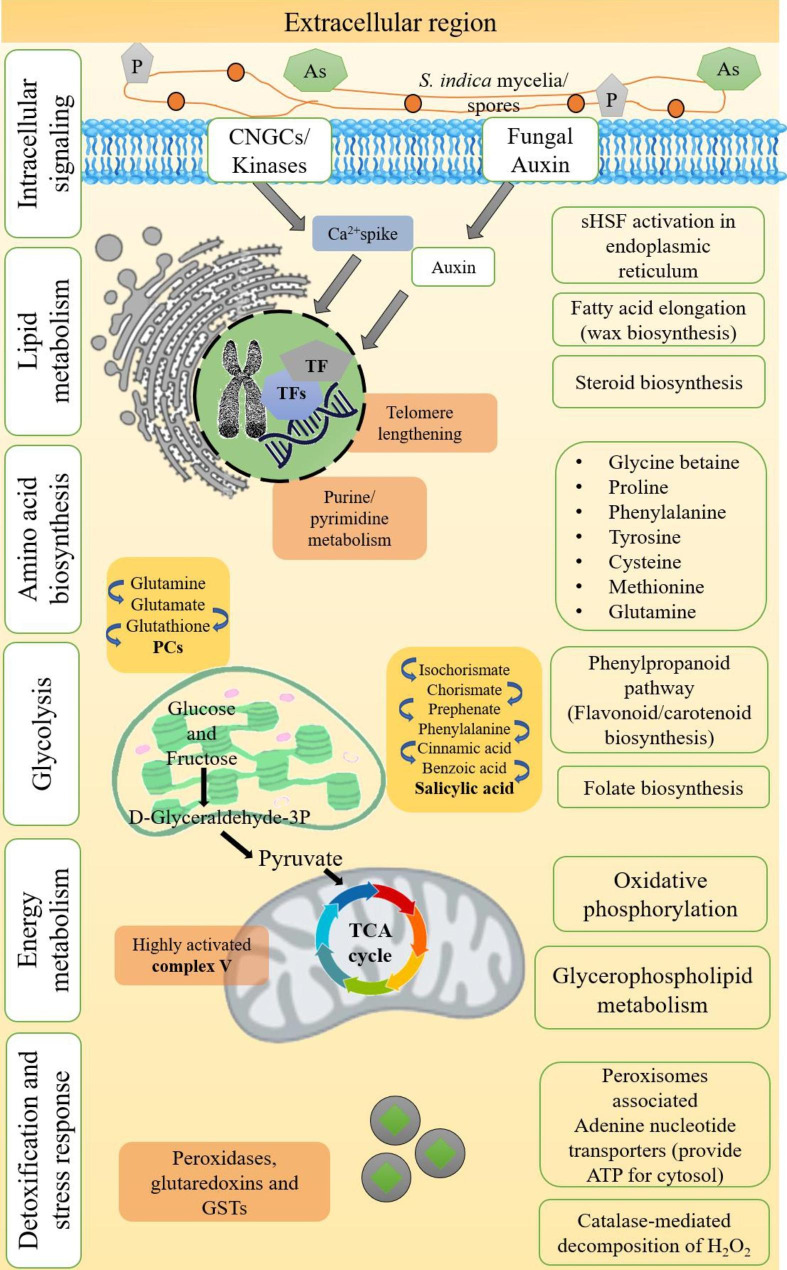
Schematic illustration of differentially expressed transcripts associated with major KEGG and GO annotated networks expressed during arsenic stress along with phosphorus fortification and *S. indica* symbiosis. These transcripts have been significantly detected when ZZY-1 under the above-mentioned treatment was compared with GD-6.

Additionally, GDSL esterase/lipase (GELPs) associated DEGs, such as *OsGELP93* (Os07g0642200), *OsGELP56* (Os04g0507700), *OsGELP67* (Os05g0419800) and *OsGELP50* (Os03g0365900) were highly expressed under T4 for GD-6. In regard to ZZY-1, the expressions were positively aggregated under T2 followed by T3, T1 and then T4. Certain cell-wall biosynthesis related genes (expansins) and different channels were expressed differentially and their expression profiles have been presented as heat map (Fig. 10 and Additional file 2: Fig. S14).

## Discussion

### The Efficient Detoxification and Exclusion of Heavy Metals

Arsenic toxicity can adversely affect the growth of a living organism by the accumulation of lipid peroxidation products and damaging the membrane integrity, however in the presence of such xenobiotics, plants minimize this damage by increasing the cellular activity of detoxification enzymes (Schroder et al. 2009). Brinke et al. (2018), in their transcriptome study on rice roots after exposure to As^III^-tainted sediments, found that a GO term “glutathione biosynthesis” was prominent. According to Verbruggen et al. (2009), glutathione biosynthesis is induced under heavy metal stress, providing a substrate for phytochelatins’ formation that constitutively participate in detoxifying several metal(loid)s. Transcripts related to ROS-detoxification were distinctly upregulated under As + P and As + *S.i* of ZZY-1 as well as T1 and T3 of GD-6. The conversion of As^V^ to As^III^ involves oxidation of glutathione (GSH) to GSSG, therefore it is not uncommon to observe glutathione-S-transferase, glutathione reductase (GR), glutaredoxin (Grx) and thioredoxin (Trx) associated transcripts being upregulated in rice roots upon As^V^ exposure (Huang et al. 2012). Arsenic stress induces lignification (Huang et al. 2012), which solidifies the cell-wall and restricts root growth and the production of ROS contribute to this As^V^-induced rigidity of the cell-wall. Genotype ZZY-1 presented a phenotype that, under the adverse As-laden growth conditions, managed to produce new root growth and adopted better avoidance strategies than GD-6. The addition of P with As led to suberization of roots internal tissues. In *Arabidopsis* roots, *ABCG2*, *ABCG6* and *ABCG20* transporters partake in endodermal suberin lamellae formation (Yadav et al. 2014). Moreover, *OsABCG5* in rice as well as *StABCG1* in potato tuber and its periderm have also been identified and designated as suberin monomer transporters (Chen et al. 2022). Moreover, the exudation of the HM-chelating organic acids, ‘malate’ and ‘citrate’ into the rhizosphere, by Al-activated malate transporter (ALMT) and multidrug and toxin extrusion (MATE) transporters, respectively, serve as a HM-resistance mechanism in plants (Liang et al. 2013; Liu & Li 2022). The transporter-facilitated secretion of organic acids not only detoxifies/extrudes toxic metal ions from roots but also improves P and Fe availability (Delhaize et al. 2012; Kobayashi and Nishizawa 2012). Transmembrane proteins, like P-type ATPases, function as pumps across the plasma or intracellular membranes, coupling ATP hydrolysis to the efflux of a cation out of the cytosol (Nuria 2021). In this study, the expression of P-type ATPase, K/Mg/Cd/Cu/Zn/Na/Ca/Na/H-transporter family protein (Os11g0485200), was found to be upregulated under all treatments of ZZY-1, showing the capability of this genotype as a strong effluxer.

### Preferential Growth of Lateral Roots and Potential Modification at Chromosome Level

Roots provide anchorage to the plants and the finer lateral roots in particular, explore the surrounding medium and forage for nutrients and water (Gutjahr et al. 2015). Given experimental conditions where As^V^ was abundant, the As tolerant plant exhibited a trait of cellular proliferation, producing lateral roots, which helped them to avoid taking up AsV, as reported in *Pterris vittata* (Wang et al. 2011), where new roots tended to accumulate and transport less As^V^ than primary roots, which on the contrary, uptake more As^III^. Generally, AMF colonization is distributed asymmetrically within the root system (Gutjahr and Paszkowski 2013). This becomes evident in rice roots that consist of three major root types, i.e., crown roots that are weakly colonized, large lateral roots that get strongly colonized, and fine lateral roots (FLRs), which are preferably not colonized (Gutjahr et al. 2009; Rebouillat et al. 2009). In current study, high number of lateral root production by the As tolerant genotype facilitated the colonization of *S. indica*, which corroborates a separate study conducted on rice employing another AMF *Rhizophagus irregularis* (Gutjahr et al. 2015). The study conducted by Choi et al. (2021), investigated the hereditary foundation of the naturally varying telomere length in *A. thaliana*, maize and rice and found a negative correlation between telomere length and flowering time. Both phosphorus nutrition and *S. indica* are reported to facilitate the transitional phase of vegetative growth towards maturity. It is not surprising that the combination of both is actually helping rice plants to reduce the flowering time by regulating the telomere lengthening and at the same time avoiding As-induced programmed cell death, as in plants the longer the length of telomere, the shorter the time taken up by plant to reach maturity, and vice versa. This knowledge of the two tested ameliorative treatments could be used in scientific development integrating different perspectives. Another aspect that differentiated the tolerant genotype from the susceptible one was the ribosomal damage repair under As alone treatment (G1T1 vs. G2T1), which if not done promptly and appropriately, would cause cellular functions to cease and programmed cell death.

### Phosphorus and ***S. indica*** Co-Treatment Induced Reprogramming

In plants, GDSL-type esterase/lipase enzymes (GELP) play key functions encompassing anther and pollen development, along with responses to biotic and abiotic stresses (Chepyshko et al. 2012; Cenci et al. 2022). A study carried out by Riemann et al. (2007), explained their response in association with light and jasmonate signaling; moreover, *OsGELP110*/*OsGELP115* were characterized as contributors to the formation of rice exine, whereas the double knockout (*osgelp110/osgelp115)* experienced male sterility (Zhang et al. 2020). Yuan et al. (2020) worked with an ER-localized GELP ‘*OsGELP34’*, and observed its essentiality in male reproduction and pollen development. Although, control plants of both genotypes displayed a certain degree of expression, arsenic alone and in combination with other treatments brought about a surge in genes related to GELP category (Additional file 2: Fig. S14), which represents the strategy of producing reproductive organs for the continuation of species.

The genotypes differed in their phytohormonal response as a large number of auxin responsive and biosynthesis related genes were upregulated under all the treatments of ZZY-1 on one hand (Additional file 2: Fig. S12), while on the other hand GD-6 predominantly experienced an upregulation of genes related to ethylene (Additional file 2: Fig. S13). We know from the results that ZZY-1 overall performed better than GD-6, which could be explained in a way that this genotype presented more plasticity due to higher auxin levels that inhibit elongation of primary root and promote formation of lateral root (Alarcón et al. 2019). It is interesting that ethylene and auxin work in synchronicity (Vanstraelen and Benková 2012). Ethylene acts as a central negative regulator of auxin levels in cells, restricting elongation, and this all happens in the root epidermis (Vaseva et al. 2018), which being in continuous contact with the environment surrounding it, encounters external cues, such as heavy metal toxicity.

In terms of nutrient transporters, phosphate-starvation related gene *OsSPX-MFS1* (Wang et al. 2012; He et al. 2021), was highly upregulated in GD-6 under As stress, which suggests that the competition of the As vs. P had much severe impact on this genotype; additionally, the control (T5), As + *S.i* + P (T4) and As + P (T2) treatments of the same genotype utilized *OsPT1*, whereas under As + *S.i* (T3) another P transporter, *OsPT2* was employed, which shows that the symbiotic association modified the phosphate uptake mechanism. Genotype ZZY-1, on the contrary, made use of only *OsPT14* under T1, T3 and T4. It is worthy to mention here that Shi et al. (2013), proposed *OsPHT2;1* (*OsPT14*) as a marker gene for screening high phosphorus efficiency genotypes, whose overexpression could increase foliar P concentrations and the biomass of rice plants. Ammonium is a preferred form of nitrogen for uptake in rice plants (Konishi and Ma 2021), and arsenic stress is known to adversely affect nitrogen metabolism, therefore it was not surprising to observe an upregulation of ammonium transporters (*OsAMT1* in case of ZZY-1 and *OsAMT2* for GD-6 along with a urea transporter *OsDUR3*), whose expression mellowed down under T4 and become somewhat closer to control values, showing that the pressure of altered ammonium nutrition was adjusted accordingly. Also, for ZZY-1 plants, the expression of the high-affinity nitrate transporter *OsNAR2.1* was upregulated under As + *S.i* and As + *S.i* + P, which is known to share a role in nitrate absorption and translocation in rice, providing plant with drought tolerance under water deficit conditions (Chen et al. 2019). Pertaining to As stress tolerance, GD-6 also had a few tricks up its sleeves, as it displayed a higher expression of *OsUGE1* (*UDP-glucose 4-epimerase 1*; Os05g0595100) under As alone stress, which is putatively involved in cell-wall carbohydrate partitioning amidst conditions where nitrogen is deficient (Guevara et al. 2014). Also, the gene expression of *OsNiR*, which encodes nitrite reductase and is indispensable for N assimilation in rice (Yu et al. 2021), was upregulated in GD-6 under all treatments, more prominently under As + P. Sulphur pool is required for the synthesis of many enzymes and antioxidant compounds, such as GSH and phytochelatins (Clemens and Ma 2016); differential upregulation of *OsSultr1* and *OsSultr3* represent the genotypic variation in regard to dealing with the As-induced disruption in S-metabolism. Abercrombie et al. (2008) demonstrated increased transcript levels of genes encoding chloroplastic and a cytosolic Cu/ZnSOD upon As^V^ exposure, which could explain the higher activity of copper and zinc transporters in this study, particularly for GD-6. Both genotypes responded in a certain way when it came to K^+^ nutrition, as numerous related genes were upregulated in both genotypes to cope with As-toxicity. Specific to ZZY-1, the expression of *OsAKT1* was induced higher under T4 than other treatments, which indicates the role of K^+^ in close connection with P against As toxicity (Ródenas et al. 2019). Having a closer look at the differential expression of metal transporters, involvement of YSL family genes, *IRT2* and *FRDL1* suggest the involvement of Fe nutrition; also, the two *NRAMP*s (natural resistance-associated macrophage proteins), i.e., *OsNramp1* and *OsNramp5* show transport activity for Fe^2+^, albeit their total Fe^2+^ uptake contribution is comparatively small (Takahashi et al. 2011; Sasaki et al. 2012). Maintenance of nutritional homeostasis is critical to plant’s growth as disruption/deficiency in one could lead to excess in the other or vice versa (Xie et al. 2019). Expressions of *OsENA1* under all As stressed treatments for ZZY-1 indicated the employment of salt stress response, as this gene in barely expresses under normal growth conditions but gets intensified under elevated salt concentrations and alkaline pH (Ruiz et al. 2003).

### Construing Stress Response and Phytohormonal Tuning

To produce functional proteins along with removing misfolded/aggregated harmful proteins, a system of protein quality control operates inside the cells that involves certain molecular chaperones, folding catalysts and proteases, which only assist and do not become a component of final products (Ellis 1993). These chaperons are categorized by their molecular-sizes, i.e., Hsp100s, Hsp90s, Hsp70s, Hsp60s, Hsp40s, and small heat-shock proteins, where each member assists in folding, refolding, oligomeric assembly, translocation, and degradation of a specific substrate proteins (Hartl et al. 2011). A large number of HSPs and related TFs were upregulated for ZZY-1 and GD-6 (under T1, T2, T3), however the excessive protein repair was reduced under T4, much more in ZZY-1. It is worth noting that *Hsp16.9 A* was upregulated mainly for ZZY-1, and higher expression of *OsHsp18* were witnessed in GD-6. These small heat shock proteins actually contribute positively to certain biotic/abiotic defense responses in rice plants (Kuang et al. 2017). According to a study conducted by Huang et al. (2012) almost 40% of the OsHsf TFs were observed to be induced in rice during As^V^ stress. In line with the stress responsive transcripts, gene expression of *PP2C1* and *NAC58* reduced in ZZY-1 compared to its control counterparts. Noteworthily, *PP2Cs* function as negative regulators of the ABA signaling network, which mediate stomatal closure (Jung et al. 2020), therefore reducing the expression of *PP2C1* could trigger ABA related signaling cascade. Additionally, the *NAC58* is a transcription activator that acts as a positive regulator of the jasmonate (JA) pathway, mediating leaf senescence and may directly regulate JA biosynthesis genes, such as *LOX2*, *AOC*, *AOS2*, *AOC1* and *OPR7* (Zhou et al. 2013). The obtained results show that most genes related to JA biosynthesis were not induced much in ZZY-1 than GD-6, rather transcript levels of salicylic acid and gibberellin biosynthesis genes were upregulated in ZZY-1 (Fig. 8). Under chilling stress, the combined application of the phytohormone salicylic acid and H_2_O_2_ raised the GA/ABA ratio, accelerating seed germination of maize (Li et al. 2017). Moreover, both genotypes under T4 showed lesser inclination towards ethylene responsive and biosynthesis genes expression (Additional file 2: Fig. S13), which suggests the differential fine tuning of phytohormonal operational mechanism, as an increase in ethylene response factors is a characteristic of arsenic toxicity in rice (Huang et al. 2012). Arsenic exposure tends to downregulate the expression of cytokinin signaling genes, such as *OsCRL4* and *OsRR20* (Huang et al. 2012), but genotype ZZY-1 displayed an upregulatory trend in the expression of *OsCRL1* under all treatments, as well as of *RR9* and *RR10* specifically under As + *S.i* + P (Fig. 9). Cytokinins are enriched in the shoot apical meristem and immature leaves, regulating nutrient sink and source activity and promote crop productivity by the activation of inflorescence meristems (Choi et al. 2011). Moreover, CK and BR interaction reportedly promotes ovule initiation and increases seed number per silique in case of *Arabidopsis* (Zu et al. 2022). Another gene worthy of mention among the stress responsive genes is *OsCIA*, which is present under normal growth conditions in panicles, leaves, and seedling tissues, however cold stress also induces its expression in anthers (Imin et al. 2006); the expressions were prominent under T1, T2 and T3 of ZZY-1, which implies that arsenic stress could also be sensed by the plants as chilling stress. ATP-binding cassette (ABC) transporters transform the energy gained from hydrolysis of ATP into trans-bilayer substrate movement either into or outside the cytoplasm (Locher 2009). Genotype GD-6, particularly under As alone stress expressed a number of ABC transporters, which indicates that in an attempt to exclude or sequester the accumulated As into the vacuoles, this genotype spent a lot of energy.

### Shift in Number of Genes Under Certain Transcriptional Factors

Among a number of TFs, FAR1, bHLH, WRKY, B3 and ERF were quite prominent, though the number of genes presented under them changed remarkably with added ameliorative treatments (Fig. 4). FAR-RED IMPAIRED RESPONSE1 (FAR1) partakes in plant growth and development processes, such as regulating phytochrome-signaling processes, branching/tillering, shade avoidance, and flowering time in *Arabidopsis thaliana* and rice (Genoud et al. 2008); in addition, it is reported by Dai et al. (2022), to positively regulate the responses to salt and temperature stress in *Eucalyptus grandis*. Similarly, ‘B3-domain’ family of transcription factors have crucial and diverse functions, such as seed growth, development and stress. The mutations of B3’s master regulators, i.e., *abi3*, *lec2*, and *fus3*, revealed the association of this TF in embryonic Fe distribution and *FERRITIN* gene expression (Grant-Grant et al. 2022).

Furthermore, a huge number of expansins were detected to be upregulated, mainly in ZZY-1 under T1, T2 and T3. The expansins mediate cell-wall loosening, and thereby, are responsible for apical growth enhancement (Choi et al. 2003), which might explain the plasticity of ZZY-1 genotype. The strategies adopted by plants against As uptake, its translocation and accumulation vary phenomenally, however prophylactic measures must be taken to ensure the safety of humans and the collective environment. Stress responses, like senescence and PCD, could be reoriented towards inflorescence/early maturity, which gives the plant a chance to reproduce under unpropitious conditions. Schematic diagram of differentially expressed transcripts associated with major KEGG and GO annotated networks expressed during arsenic stress along with phosphorus fortification and *S. indica* symbiosis is illustrated in Fig. 10.

## Conclusions

Arsenic toxicity to the plants can be reduced by using self-renewing and cost-effective methods, such as AMF incorporation, which is less intrusive and potentially delays phosphate starvation response with adequate P supply. This study showed that the As + *S.i* + P interactive treatment mellowed down the chaotic increase in heat shock proteins, and potentially induced early flowering genes by lengthening the telomere. Keeping the above information into consideration, genes stimulating endogenous BR (*CycD3*) and their cross-talk with CK (*Os11g0143300*, *Os12g0139400*, *Os01g0952500* and *Os01g0197700*), as well as SA (*Os11g0663100*, *Os03g0778000* and *Os11g0669100*), metal effluxer (P-type ATPase, K/Mg/Cd/Cu/Zn/Na/Ca/Na/H-transporter family protein) and detoxification related genes (glutathione-S-transferases), elevation of K^+^ transporter *AKT1*, Fe^+^ transporters (*OsYSL2* and *OsYSL15*), and phosphate transporter *PHT14*, greatly contributed to As tolerance acquisition. Supply of P remarkably changed the response of plants colonized with *S. indica*, which raises questions regarding the efficiency of this AMF and the untapped potential that could be exploited by combining this tool with other HM remediation techniques.

## Electronic Supplementary Material

Below is the link to the electronic supplementary material.

**Additional file 1:Table S1**. List of salts used in making nutrient solution. **Table S2**. Sequences of oligonucleotide primers used for validating RNA-Seq data. **Table S3**. Summary statistics of rice transcriptome generated by Illumina NovaSeq 6000. **Table S4**. List of novel genes detected during As/*S. indica*/P interaction in ZZY-1 and GD-6. **Table S5**. Inclusively expressed detoxification-related differential transcripts (log_2_ fold change) observed in response to As/*S. indica*/P interaction in ZZY-1 and GD-6. **Table S6**. Inclusively expressed nutrient and metal transport-related differential transcripts (log_2_ fold change) observed in response to As/*S. indica*/P interaction in ZZY-1 and GD-6. **Table S7**. Inclusively expressed phytohormone-related differential transcripts (log_2_ fold change) observed in response to As/*S. indica*/P interaction in ZZY-1 and GD-6.

**Additional file 2:Fig. S1**. Validation of data obtained from RNA-Seq with real-time quantitative reverse transcription PCR. **Fig. S2**. Summary of gene mapping ratio as observed across the samples including all treatments between ZZY-1 (G1) and GD-6 (G2). **Fig. S3**. Venn diagram representing exclusive and inclusive DEGs expressed for G1 = ZZY1 (A), and G2 = GD-6 (B), in response to As alone, As + P, As + *S.i* and As + *S.i* + P treatments along with control. **Fig. S4**. Principal component analysis (PCA) of As, P and *S. indica*-induced transcripts across the treatments in two rice genotypes. **Fig. S5**. Time series analysis illustrating Mfuzz results. T1 = As_10__µM_, T2 = As_10__µM_ + P_50µM_, T3 = As_10__µM_ + *S.i*, T4 = As_10__µM_ + *S.i* + P_50__µM_ and T5 = control. **Fig. S6**. Distribution of differentially expressed transcripts using color module (A), and cluster dendrogram (B). **Fig. S7**. KEGG analysis of DEGs presented as scatter plot showing 20 most significantly enriched pathways for: (A) G1T1 vs. G2T1, (B) G1T2 vs. G2T2, and (C) G1T3 vs. G2T3 comparisons. **Fig. S8**. Relative comparative gene ontology (GO) in ZZY-1 under As_10_ (A), As_10__µM_ + P_50µM_ (B), and As_10__µM_ + *S.i* (C) in comparison to GD-6. **Fig. S9**. Heat map presenting the expression profile of mutually expressed stress responsive DEGs. **Fig. S10**. Heat map showing the expression profile of inclusively expressed heavy metal associated transporters. **Fig. S11**. Heat map representing the expression profile of inclusively expressed ABC transporters. **Fig. S12**. Hierarchical heat map depicting the expression profile of inclusively expressed genes related to auxin biosynthesis and signaling. **Fig. S13**. Hierarchical heat map illustrating the expression profile of inclusively expressed genes related to the biosynthesis and signaling of ethylene. **Fig. S14**. Hierarchical heatmap representation of differentially expressed *GELP* transcripts (inclusively/exclusively) observed in response to As alone, As + P, As + *S.i* and As + *S.i* + P along with control, in two rice genotypes.

## Data Availability

All data generated or analysed during this study are included in this published article [and its supplementary information files].
