# Supplementary material for: Pan-transcriptomic Profiling Demarcates Serendipita Indica-Phosphorus Mediated Tolerance Mechanisms in Rice Exposed to Arsenic Toxicity
Source: Rice (N Y). 2023 Jun 24;16:28. doi: 10.1186/s12284-023-00645-0 (PMC10290630; doi:10.1186/s12284-023-00645-0)
Supplement: Supplementary file 1 — Additional file 1:Table S1. List of salts used in making nutrient solution. Table S2. Sequences of oligonucleotide primers used for validating RNA-Seq data. Table S3. Summary statistics of rice transcriptome generated by Illumina NovaSeq 6000. Table S4. List of novel genes detected during As/S. indica/P interaction in ZZY-1 and GD-6. Table S5. Inclusively expressed detoxification-related differential transcripts (log2 fold change) observed in response to As/S. indica/P interaction in ZZY-1 and GD-6. Table S6. Inclusively expressed nutrient and metal transport-related differential transcripts (log2 fold change) observed in response to As/S. indica/P interaction in ZZY-1 and GD-6. Table S7. Inclusively expressed phytohormone-related differential transcripts (log2 fold change) observed in response to As/S. indica/P interaction in ZZY-1 and GD-6. [file 12284_2023_645_MOESM1_ESM.docx]

**Pan-transcriptomic profiling demarcates *Serendipita indica*-phosphorus mediated tolerance mechanisms in rice exposed to arsenic toxicity**

Shafaque Sehar ^1†^, Muhammad Faheem Adil^1†^, Syed Muhammad Hassan Askri^1^, Qidong Feng^1^, Dongming Wei^1^, Falak Sehar Sahito^2^, Imran Haider Shamsi^1*^

^1^Zhejiang Key Laboratory of Crop Germplasm Resource, Department of Agronomy, College of Agriculture and Biotechnology, Zhejiang University, Hangzhou 310058, China

^2^ Dow International Medical College, Dow University of Health Sciences, Karachi 74200, Pakistan

*Corresponding author’s email: [drimran@zju.edu.cn](mailto:drimran@zju.edu.cn)

^†^These authors contributed equally to this study

**Table S1.** List of salts used in making nutrient solution.

| **Compound name** | **Concentrations** (L^−1^**)** |
| --- | --- |
| Ammonium nitrate \| NH_4_NO_3_ | 2.9 mmol |
| Magnesium sulfate heptahydrate \| MgSO_4_.7H_2_O | 1.7 mmol |
| Potassium Sulfate \| K_2_SO_4_ | 1.0 mmol |
| Calcium chloride \| CaCl_2_ | 1.0 mmol |
| Sodium dihydrogen phosphate dehydrate \| NaH_2_PO_4_·2H_2_O | 0.125 mmol |
| EDTA iron(III) sodium salt \| EDTAFeNa | 36 µmol |
| Boric Acid \| H_3_BO_3_ | 18 µmol |
| Manganese (II) chloride tetrahydrate \| MnCl_2_.4H_2_O | 9.1 µmol |
| Ammonium molybdate tetrahydrate \| NH_4_)_6_Mo_7_O_24_.4H_2_O | 0.52 µmol |
| Copper sulfate pentahydrate \| CuSO_4_.5H_2_O | 0.16 µmol |
| Zinc sulfate heptahydrate \| ZnSO_4_.7H_2_O | 0.15 µmol |

**Table S2**. Sequences of oligonucleotide primers used for validating RNA-Seq data.

| Gene-ID | Gene-name | Forward (5' to 3') | Reverse (5' to 3') |
| --- | --- | --- | --- |
| Os09g0478100 | *OsCslE1* | CCTGCTTGGTAAGGGTCGTT | AATCAAGCGCTCACTGCTCA |
| Os06g0726200 | *OsCht1* | CTACTGTGTCCAGAGCTCGC | TTTCCATCAGCCATTGTGGG |
| Os11g0216900 | *OsIDI2* | ACTGCCTTGGGGGTTATTCG | AGCACTCTACGATCCTCCCT |
| Os02g0301100 | *OsSWEET4* | ATGCATGTGTGTGACTGCCT | GAAAGAAACGGATGGCGACAC |
| Os05g0415200 | *OsPCS1* | TCGCTTCAAATACCCTCCTC | TTTACTTGGGCTGGATCCTC |
| Os01g0952800 | *OsIRO2* | CTTGAGGCGGACATCTTCTC | AGAGCTCGTTGAGCTGCTTC |
| Os03g0216700 | *OsFRDL1* | AGCAGCCAAGATCTGGAGAA | AGACCAGGAAGACAGCCTGA |
| Os02g0649900 | *OsYSL2* | AGAACACCGTTGTCCAGACC | TAAGGAGCCCAACGAAGCTA |
| Os05g0111300 | *OsMT2b* | AAGAAGCCTGGCACGCATGAG | TGCGTGTGTCGATCAATGTTGGA |

**Table S3.** Summary statistics of rice transcriptome generated by Illumina NovaSeq 6000.

| Sample name | Raw reads | Total/Clean reads | Total  mapped | Clean bases | Multiple mapped | Unique  mapped | Q20 (%) | Q30  (%) | GC  (%) |
| --- | --- | --- | --- | --- | --- | --- | --- | --- | --- |
| G1T1R1 | 55156662 | 55156570 | 45189751(81.93%) | 8.26G | 1106261(2.01%) | 44083490(79.92%) | 97.38 | 91.67 | 50.61 |
| G1T1R2 | 58193722 | 58193624 | 48347611(83.08%) | 8.72G | 1338831(2.30%) | 47008780(80.78%) | 97.9 | 93.23 | 50.34 |
| G1T1R3 | 53600630 | 53600558 | 45138175(84.21%) | 8.02G | 1334819(2.49%) | 43803356(81.72%) | 97.84 | 93.02 | 50.75 |
| G1T2R1 | 59933438 | 59933252 | 51379742(85.73%) | 8.97G | 1273071(2.12%) | 50106671(83.60%) | 97.56 | 92.56 | 50.35 |
| G1T2R2 | 57029170 | 57029036 | 51275566(89.91%) | 8.54G | 1385320(2.43%) | 49890246(87.48%) | 98.07 | 93.83 | 50.79 |
| G1T2R3 | 51966796 | 51966730 | 45821355(88.17%) | 7.77G | 1251714(2.41%) | 44569641(85.77%) | 97.59 | 92.29 | 50.57 |
| G1T3R1 | 62515380 | 62515314 | 56362277(90.16%) | 9.36G | 1635529(2.62%) | 54726748(87.54%) | 97.92 | 93.33 | 50.72 |
| G1T3R2 | 58885560 | 58885494 | 46563575(79.07%) | 8.82G | 1250975(2.12%) | 45312600(76.95%) | 97.66 | 92.46 | 50.65 |
| G1T3R3 | 58514814 | 58514744 | 44682326(76.36%) | 8.76G | 1151143(1.97%) | 43531183(74.39%) | 97.55 | 92.08 | 51.47 |
| G1T4R1 | 47419802 | 47419736 | 41040201(86.55%) | 7.09G | 1016474(2.14%) | 40023727(84.40%) | 98.01 | 93.62 | 50.09 |
| G1T4R2 | 65091784 | 65091676 | 45253649(69.52%) | 9.73G | 1106734(1.70%) | 44146915(67.82%) | 98.45 | 94.93 | 46.91 |
| G1T4R3 | 62699186 | 62699122 | 50878819(81.15%) | 9.38G | 1361381(2.17%) | 49517438(78.98%) | 97.9 | 93.23 | 50.13 |
| G1T5R1 | 66762088 | 66762004 | 51905652(77.75%) | 9.96G | 1266461(1.90%) | 50639191(75.85%) | 97.76 | 92.86 | 49.99 |
| G1T5R2 | 52232056 | 52231976 | 40193979(76.95%) | 7.77G | 1057196(2.02%) | 39136783(74.93%) | 97.77 | 92.81 | 50.36 |
| G1T5R3 | 65163816 | 65163762 | 54548939(83.71%) | 9.75G | 1354423(2.08%) | 53194516(81.63%) | 97.6 | 92.27 | 51.33 |
| G2T1R1 | 72243860 | 72243712 | 60009656(83.07%) | 10.78G | 1541164(2.13%) | 58468492(80.93%) | 98.05 | 93.75 | 50.98 |
| G2T1R2 | 60029088 | 60029018 | 48603105(80.97%) | 8.97G | 1260892(2.10%) | 47342213(78.87%) | 97.54 | 92.15 | 51.13 |
| G2T1R3 | 65331334 | 65331234 | 46181411(70.69%) | 9.77G | 1216888(1.86%) | 44964523(68.83%) | 97.49 | 91.94 | 51.42 |
| G2T2R1 | 63203312 | 63203190 | 54246628(85.83%) | 9.44G | 1438899(2.28%) | 52807729(83.55%) | 97.6 | 92.3 | 51.66 |
| G2T2R2 | 60807076 | 60806996 | 51519384(84.73%) | 9.09G | 1390045(2.29%) | 50129339(82.44%) | 97.38 | 91.63 | 51.38 |
| G2T2R3 | 65214578 | 65214498 | 51271617(78.62%) | 9.76G | 1366808(2.10%) | 49904809(76.52%) | 97.62 | 92.43 | 51.66 |
| G2T3R1 | 63809750 | 63809624 | 46152938(72.33%) | 9.53G | 1104539(1.73%) | 45048399(70.60%) | 97.58 | 92.28 | 51.16 |
| G2T3R2 | 60889308 | 60889224 | 44271923(72.71%) | 9.1G | 1050291(1.72%) | 43221632(70.98%) | 97.68 | 92.51 | 50.98 |
| G2T3R3 | 52021874 | 52021818 | 38979246(74.93%) | 7.79G | 1004975(1.93%) | 37974271(73.00%) | 97.61 | 92.18 | 51.61 |
| G2T4R1 | 58883054 | 58882974 | 41247722(70.05%) | 8.8G | 960058(1.63%) | 40287664(68.42%) | 97.59 | 92.26 | 49.86 |
| G2T4R2 | 61876778 | 61876660 | 48194693(77.89%) | 9.24G | 1133949(1.83%) | 47060744(76.06%) | 98.01 | 93.63 | 50.58 |
| G2T4R3 | 86720224 | 86052530 | 61877406(71.91%) | 12.88G | 2268813(2.64%) | 59608593(69.27%) | 97.61 | 92.52 | 51.28 |
| G2T5R1 | 46351992 | 46351934 | 37687625(81.31%) | 6.92G | 916694(1.98%) | 36770931(79.33%) | 97.8 | 92.95 | 50.43 |
| G2T5R2 | 53045128 | 53045056 | 44166633(83.26%) | 7.93G | 1076384(2.03%) | 43090249(81.23%) | 97.68 | 92.6 | 50.7 |
| G2T5R3 | 50084352 | 50084288 | 40669402(81.20%) | 7.49G | 1024314(2.05%) | 39645088(79.16%) | 97.92 | 93.35 | 50.4 |

**Table S4.** List of novel genes detected during As/*S. indica*/P interaction in ZZY-1 and GD-6.

| Gene-ID | GO-ID | GO category | Putative function |
| --- | --- | --- | --- |
| Novel.10674 | GO:0008137 | MF | NADH dehydrogenase (ubiquinone) activity |
| Novel.10700 | GO:0003735 | MF | Structural constituent of ribosome |
| Novel.17783 | GO:0020037 | MF | Heme binding |
| Novel.17885 | GO:0003824 | MF | Catalytic activity |
| Novel.18755 | GO:0015095 | MF | Magnesium ion transmembrane transporter activity |
| Novel.21814 | GO:0003677 | MF | DNA binding |
| Novel.24621 | GO:0015288 | MF | Porin activity |
| Novel.25513 | GO:0051538 | MF | 3 iron, 4 sulfur cluster binding |
| Novel.25513 | GO:0050660 | MF | Flavin adenine dinucleotide binding |
| Novel.25513 | GO:0005506 | MF | Iron ion binding |
| Novel.25513 | GO:0010181 | MF | FMN binding |
| Novel.25513 | GO:0016040 | MF | Glutamate synthase (NADH) activity |
| Novel.28605 | GO:0015081 | MF | Sodium ion transmembrane transporter activity |
| Novel.30957 | GO:0015254 | MF | Glycerol channel activity |
| Novel.30957 | GO:0015250 | MF | Water channel activity |
| Novel.32378 | GO:0003735 | MF | Structural constituent of ribosome |
| Novel.32378 | GO:0004807 | MF | Triose-phosphate isomerase activity |
| Novel.33188 | GO:0003723 | MF | RNA binding |
| Novel.36009 | GO:0004386 | MF | Helicase activity |
| Novel.36009 | GO:0016887 | MF | ATPase activity |
| Novel.36009 | GO:0003677 | MF | DNA binding |
| Novel.36009 | GO:0005524 | MF | ATP binding |
| Novel.36009 | GO:0031491 | MF | Nucleosome binding |
| Novel.4057 | GO:0005524 | MF | ATP binding |
| Novel.4057 | GO:0004674 | MF | Protein serine/threonine kinase activity |
| Novel.5572 | GO:0020037 | MF | Heme binding |
| Novel.5573 | GO:0003735 | MF | Structural constituent of ribosome |
| Novel.5580 | GO:0003735 | MF | Structural constituent of ribosome |
| Novel.5580 | GO:0019843 | MF | rRNA binding |
| Novel.8003 | GO:0005516 | MF | Calmodulin binding |
| Novel.8003 | GO:0005524 | MF | ATP binding |
| Novel.8408 | GO:0016984 | MF | Ribulose-bisphosphate carboxylase activity |
| Novel.8408 | GO:0000287 | MF | Magnesium ion binding |
| Novel.10674 | GO:0016021 | CC | Integral component of membrane |
| Novel.10674 | GO:0005739 | CC | Mitochondrion |
| Novel.10700 | GO:0015934 | CC | Large ribosomal subunit |
| Novel.10700 | GO:0009507 | CC | Chloroplast |
| Novel.17783 | GO:0016021 | CC | Integral component of membrane |
| Novel.17783 | GO:0009535 | CC | Chloroplast thylakoid membrane |
| Novel.18755 | GO:0016021 | CC | Integral component of membrane |
| Novel.23303 | GO:0016021 | CC | Integral component of membrane |
| Novel.24621 | GO:0005741 | CC | Mitochondrial outer membrane |
| Novel.24621 | GO:0046930 | CC | Pore complex |
| Novel.25513 | GO:0009507 | CC | Chloroplast |
| Novel.28605 | GO:0005886 | CC | Plasma membrane |
| Novel.30957 | GO:0009705 | CC | Plant-type vacuole membrane |
| Novel.30957 | GO:0005887 | CC | Integral component of plasma membrane |
| Novel.30957 | GO:0042807 | CC | Central vacuole |
| Novel.32378 | GO:0005840 | CC | Ribosome |
| Novel.32378 | GO:0009507 | CC | Chloroplast |
| Novel.33188 | GO:0009507 | CC | chloroplast |
| Novel.3559 | GO:0005739 | CC | Mitochondrion |
| Novel.36009 | GO:0016589 | CC | NURF complex |
| Novel.5572 | GO:0016021 | CC | Integral component of membrane |
| Novel.5572 | GO:0009535 | CC | Chloroplast thylakoid membrane |
| Novel.5573 | GO:0015934 | CC | Large ribosomal subunit |
| Novel.5573 | GO:0009507 | CC | Chloroplast |
| Novel.5580 | GO:0005840 | CC | Ribosome |
| Novel.5580 | GO:0009507 | CC | chloroplast |
| Novel.8003 | GO:0033588 | CC | Elongator holoenzyme complex |
| Novel.8003 | GO:0005737 | CC | Cytoplasm |
| Novel.8003 | GO:0005634 | CC | Nucleus |
| Novel.8408 | GO:0009507 | CC | Chloroplast |
| Novel.10674 | GO:0055114 | BP | Oxidation-reduction process |
| Novel.10700 | GO:0006412 | BP | Translation |
| Novel.17783 | GO:0017004 | BP | Cytochrome complex assembly |
| Novel.18755 | GO:1903830 | BP | Magnesium ion transmembrane transport |
| Novel.18755 | GO:0015693 | BP | Magnesium ion transport |
| Novel.24621 | GO:0055085 | BP | Transmembrane transport |
| Novel.24621 | GO:0006811 | BP | Ion transport |
| Novel.25513 | GO:0019676 | BP | Ammonia assimilation cycle |
| Novel.25513 | GO:0055114 | BP | Oxidation-reduction process |
| Novel.25513 | GO:0097054 | BP | L-glutamate biosynthetic process |
| Novel.28605 | GO:0035725 | BP | Sodium ion transmembrane transport |
| Novel.30957 | GO:0009992 | BP | Cellular water homeostasis |
| Novel.30957 | GO:0006833 | BP | Water transport |
| Novel.30957 | GO:0015793 | BP | Glycerol transport |
| Novel.30957 | GO:0034220 | BP | Ion transmembrane transport |
| Novel.32378 | GO:0006412 | BP | translation |
| Novel.32378 | GO:0006096 | BP | Glycolytic process |
| Novel.33188 | GO:0008380 | BP | RNA splicing |
| Novel.33188 | GO:0006397 | BP | mRNA processing |
| Novel.33188 | GO:0008033 | BP | tRNA processing |
| Novel.36009 | GO:0043044 | BP | ATP-dependent chromatin remodeling |
| Novel.4057 | GO:0006468 | BP | Protein phosphorylation |
| Novel.5572 | GO:0017004 | BP | Cytochrome complex assembly |
| Novel.5573 | GO:0006412 | BP | Translation |
| Novel.5580 | GO:0006412 | BP | Translation |
| Novel.5580 | GO:0000027 | BP | Ribosomal large subunit assembly |
| Novel.8003 | GO:0080178 | BP | 5-carbamoylmethyl uridine residue modification |
| Novel.8003 | GO:0006357 | BP | Regulation of transcription by RNA polymerase II |
| Novel.8408 | GO:0015979 | BP | Photosynthesis |
| Novel.8408 | GO:0015977 | BP | Carbon fixation |

**Table S5**. Inclusively expressed detoxification-related differential transcripts (log_2_ fold change) observed in response to As/*S. indica*/P interaction in ZZY-1 and GD-6.

| Gene ID | G1T1 *vs.* G2T1 | G1T2 *vs.* G2T2 | G1T3 *vs.* G2T3 | G1T4 *vs.* G2T4 | Description |
| --- | --- | --- | --- | --- | --- |
| Os02g0813500 | -1.0967 | - | - | - | *OsGR2* |
| Os03g0135300 | 1.1928 | - | - | - | *OsGSTF3* |
| Os06g0168000 | 1.0223 | - | - | - | GST, C-terminal-like domain containing protein |
| Os06g0185966 | -1.8261 | - | - | - | Similar to Glutathione peroxidase |
| Os01g0933900 | - | -1.5017 | - | - | *OsGSTF4* |
| Os10g0530600 | - | 1.7561 | - | - | *OsGSTU48* |
| Os03g0134900 | - | 1.685 | - | - | *OsGSTF14* |
| Os01g0949700 | - | -1.0272 | - | - | *OsGSTU7* |
| Os10g0415300 | - | 1.0047 | - | - | *OsGR3* |
| Os10g0543800 | - | 1.7697 | - | - | *OsRPP31* |
| Os03g0135100 | - | 1.8822 | - | - | *OsGSTF15* |
| Os06g0227500 | - | 1.0465 | - | - | *OsGSTU38* |
| Os01g0371500 | - | -1.378 | - | - | *OsGSTF10* |
| Os10g0530400 | - | -1.7799 | - | - | *OsGSTU23* |
| Os12g0263000 | - | - | 1.1906 | - | Similar to Glutathione synthase |
| Os12g0263050 | - | - | 1.0153 | - | Similar to Glutathione synthase |
| Os10g0527601 | - | - | - | -1.6751 | *OsGSTU20* |
| Os01g0692100 | - | - | - | -2.0973 | *OsGSTU39* |
| Os10g0525500 | - | - | - | -1.1171 | *OsGSTU21* |
| Os04g0556300 | - | - | - | -1.214 | *OsGpx1* |
| Os11g0284900 | - | - | - | -2.2701 | *OsGpx5* |
| Os01g0371400 | - | - | - | 3.0144 | *OsGSTF9* |
| Os05g0412800 | - | - | - | 1.3693 | *OsGSTU16* |
| Os01g0667900 | - | -1.5786 | - | - | *GRX6* |
| Os05g0198200 | - | - | -1.0784 | - | *OsGRX16* |
| Os06g0695200 | -2.0076 | - | - | - | *prx93* |
| Os04g0688200 | 2.2995 | - | - | - | *prx60* |
| Os12g0178100 | 1.0449 | - | - | - | *OsAPx6* |
| Os01g0962900 | -4.5187 | - | - | - | *prx21* |
| Os07g0604550 | -1.0871 | - | - | - | Mitochondrial inner membrane translocase subunit Tim17/Tim22/Tim23 |
| Os01g0205900 | 4.8403 | - | - | - | *prx2* |
| Os03g0805400 | -1.1426 | - | - | - | Phosphatidic acid phosphatase type 2/haloperoxidase domain containing protein |
| Os01g0159400 | -1.1244 | - | - | - | Similar to Acyl-coenzyme A oxidase 4, peroxisomal (EC 1.3.3.6) (AOX 4) |
| Os06g0625500 | 1.0215 | - | - | - | *OsPrxII* |
| Os05g0163700 | -1.2544 | - | - | - | *OsACX4* |
| Os04g0689000 | 3.5007 | - | - | - | *prx64* |
| Os03g0339400 | - | 1.6519 | - | - | *prx40* |
| Os06g0143000 | - | -1.1874 | - | - | *SodB* |
| Os01g0172600 | - | -1.5053 | - | - | Similar to electron carrier/ heme binding / peroxidase |
| Os01g0263000 | - | 1.5562 | - | - | *prx5* |
| Os12g0112000 | - | 1.7856 | - | - | *Pox* |
| Os04g0623600 | - | -1.2273 | - | - | *GLO2* |
| Os01g0787000 | - | -1.1512 | - | - | *prx19* |
| Os04g0608600 | - | -1.3002 | - | - | Alkyl hydroperoxide reductase subunit C (AhpC) |
| Os07g0639400 | - | -1.5939 | - | - | *prx108* |
| Os01g0327400 | - | -1.0662 | - | - | *prx16* |
| Os06g0196300 | - | -2.1938 | - | - | *OsPrx* |
| Os02g0161800 | - | 2.1064 | - | - | *prx24* |
| Os07g0104233 | - | -4.2859 | - | - | Similar to peroxidase 1 |
| Os06g0695300 | - | -2.8968 | - | - | *prx92* |
| Os04g0223300 | - | 1.4024 | - | - | *OsAPx3* |
| Os08g0522400 | - | 1.1682 | - | - | Similar to APX6; L-ascorbate peroxidase/ heme binding / peroxidase |
| Os03g0339300 | - | 4.2517 | - | - | *PRX41* |
| Os05g0134400 | - | 1.8595 | - | - | *prx65* |
| Os09g0471100 | - | -2.2184 | - | - | *prx122* |
| Os09g0323700 | - | - | -4.3162 | - | *prx121* |
| Os12g0191500 | - | - | -3.6461 | - | *prx137* |
| Os05g0135200 | - | - | 1.7507 | - | *prx69* |
| Os03g0368300 | - | - | 2.5719 | - | *prx43* |
| Os03g0368000 | - | - | 3.8726 | - | *PRX42* |
| Os04g0651000 | - | - | -1.4909 | - | *prx57* |
| Os01g0284500 | - | - | 1.4988 | - | *GER4* |
| Os08g0113000 | - | - | -1.3705 | - | *prx117* |
| Os07g0639000 | - | - | 2.2451 | - | *prx107* |
| Os12g0111800 | - | - | -1.2633 | - | Similar to Class III peroxidase 136 |
| Os03g0235000 | - | - | -1.1355 | - | *poxN* |
| Os06g0306300 | - | - | 1.9388 | - | *prx78* |
| Os03g0434800 | - | - | 4.2019 | - | Similar to Peroxidase 2 |
| Os01g0294700 | - | - | 1.7027 | - | *OsPOD* |
| Os05g0162000 | - | - | - | -1.1911 | *prx72* |
| Os07g0677100 | - | - | - | -2.3189 | *POX8.1* |
| Os07g0638300 | - | - | - | -1.2925 | Os1-CysPrxA |
| Os01g0327100 | - | - | - | -1.0346 | *prx15* |
| Os02g0192700 | - | - | - | -1.2793 | Thioredoxin peroxidase |
| Os07g0638600 | - | - | - | 4.0337 | *prx105* |
| Os06g0546500 | - | - | - | -1.2146 | *prx88* |
| Os02g0240100 | - | - | - | -2.8986 | *prx30* |

Treatments were denoted as: T1=As_10µM_, T2=As_10µM_+P_50µM_, T=3As_10µM_+*S.i*, T4=As+*S.i*+P_50µM_.

**Table S6.** Inclusively expressed nutrient and metal transport-related differential transcripts (log_2_ fold change) observed in response to As/*S. indica*/P interaction in ZZY-1 and GD-6.

| Gene ID | G1T1 *vs.* G2T1 | G1T2 *vs.* G2T2 | G1T3 *vs.* G2T3 | G1T4 *vs.* G2T4 | Description |
| --- | --- | --- | --- | --- | --- |
| Os01g0290700 | 1.1507 | - | - | - | *OsABCB1* |
| Os04g0642000 | 1.9586 | - | - | - | *OsABCB16* |
| Os05g0548500 | -1.2591 | - | - | - | *OsABCB19* |
| Os11g0177400 | 1.4351 | - | - | - | *OsABCG27* |
| Os08g0544400 | -2.0712 | - | - | - | *OsABCG45* |
| Os04g0627200 | 1.2067 | - | - | - | *OsABCI1* |
| Os01g0770500 | 1.1904 | - | - | - | *OsABCI6* |
| Os09g0333600 | -4.6534 | - | - | - | PDR-like ABC transporter (PDR4 ABC transporter) |
| Os06g0589300 | - | -1.2778 | - | - | *OsABCA2* |
| Os08g0398300 | - | -1.4241 | - | - | *OsABCA4* |
| Os04g0481700 | - | 3.0933 | - | - | *OsABCB15* |
| Os05g0137200 | - | 1.8476 | - | - | *OsABCB17* |
| Os04g0588700 | - | -1.06 | - | - | *OsABCC7* |
| Os02g0107900 | - | 2.073 | - | - | ABC transporter family, cholesterol/phospholipid flippase |
| Os02g0211000 | - | - | -1.2747 | - | *OsABCA1* |
| Os08g0398350 | - | - | 1.1835 | - | *OsABCA5* |
| Os02g0693700 | - | - | 2.7936 | - | *OsABCB11* |
| Os06g0158900 | - | - | 1.2327 | - | *OsABCC15* |
| Os11g0416900 | - | - | -1.4747 | - | *OsABCG28* |
| Os01g0695800 | - | - | - | -1.1509 | *MDR4* |
| Os05g0548300 | - | - | - | 1.4315 | *OsABCB18* |
| Os01g0121600 | - | - | - | -1.2728 | *OsABCG1* |
| Os09g0445600 | 1.5484 | - | - | - | Similar to oxidoreductase/ transition metal ion binding protein |
| Os03g0120400 | 1.006 | - | - | - | Heavy metal transport/detoxification protein domain containing protein |
| Os07g0274100 | - | -1.585 | - | - | Metallophosphoesterase domain containing protein |
| Os04g0667600 | - | -1.7807 | - | - | HM transport/detoxification protein domain containing protein |
| Os03g0111400 | - | 1.3175 | - | - | HM transport/detoxification protein domain containing protein |
| Os02g0582600 | - | -1.3059 | - | - | HM transport/detoxification protein domain containing protein |
| Os01g0842600 | - | 1.1565 | - | - | *OsFtsH3* |
| Os08g0512200 | - | 2.3261 | - | - | pi55-2 HM-associated domain containing protein |
| Os08g0557200 | - | - | 3.2437 | - | Metallophosphoesterase domain containing protein |
| Os01g0125600 | - | - | 1.2122 | - | HM transport/detoxification protein domain containing protein |
| Os01g0881400 | - | - | -1.1339 | - | DNA repair metallo-beta-lactamase domain containing protein |
| Os07g0682000 | - | - | 1.7624 | - | HM transport/detoxification protein domain containing protein |
| Os08g0403300 | - | - | -2.216 | - | HM transport/detoxification protein domain containing protein |
| Os10g0209700 | - | - | -1.1638 | - | HM transport/detoxification protein domain containing protein |
| Os11g0549615 | - | - | -2.365 | - | *OsPAP3a* |
| Os07g0298900 | - | - | - | 2.1886 | *OsEnS-104* |
| Os01g0678800 | - | - | - | -1.1662 | HM transport/detoxification protein domain containing protein |
| Os04g0244800 | - | - | - | -1.0213 | HM transport/detoxification protein domain containing protein |
| Os07g0516600 | 1.6999 | - | - | - | Multi antimicrobial extrusion (MatE) protein |
| Os02g0676400 | 1.6935 | - | - | - | Multi antimicrobial extrusion (MatE) protein |
| Os06g0707100 | - | -1.1946 | - | - | Multi antimicrobial extrusion (MatE) protein |
| Os08g0480000 | - | 1.0185 | - | - | Multi antimicrobial extrusion (MatE) protein |
| Os08g0545900 | - | 3.6235 | - | - | Multi antimicrobial extrusion (MatE) protein |
| Os06g0495500 | - | - | 1.0787 | - | Multi antimicrobial extrusion (MatE) protein |
| Os03g0626700 | - | - | - | -1.3938 | Multi antimicrobial extrusion (MatE) protein |
| Os03g0638200 | -1.1245 | - | - | - | Similar to Major facilitator superfamily (MFS) protein |
| Os12g0133100 | - | 1.3257 | - | - | *OsZIFL12* |
| Os07g0180700 | - | -1.8779 | - | - | *OsZIFL3* |
| Os11g0135000 | - | - | -1.1214 | - | *OsZIFL5* |
| Os01g0279400 | - | - | 1.6071 | - | *OsZIFL2* |
| Os12g0180100 | - | - | 2.0416 | - | *OsPT26* |
| Os10g0109900 | - | - | 2.8096 | - | Major facilitator superfamily, general substrate transporter domain containing protein |
| Os03g0168000 | - | - | 1.2449 | - | Major facilitator superfamily, general substrate transporter domain containing protein |
| Os03g0178100 | - | -1.5189 | - | - | *OsHMA6* |
| Os06g0700700 | - | -1.0013 | - | - | *HMA2* |
| Os09g0408550 | - | 1.0553 | - | - | Heavy metal-associated domain, HMA domain containing protein |
| Os01g0249800 | - | -2.8504 | - | - | Heavy metal-associated domain, HMA domain containing protein |
| Os07g0232900 | - | -1.2061 | - | - | *HMA3* |
| Os04g0390500 | -1.0343 | - | - | - | *OsYSL6* |
| Os02g0116300 | - | - | 1.2736 | - | *OsYSL7* |
| Os04g0524900 | - | - | 1.6626 | - | *OsYSL11* |
| Os04g0542800 | - | - | -1.5608 | - | *YSL16* |
| Os07g0155600 | - | -1.15 | - | - | Nramp ion-transporter family protein |
| Os02g0131800 | - | - | 1.8836 | - | NRAMP4, Trivalent AI influx transporter, Aluminum (Al) tolerance |
| Os03g0216700 | - | -1.513 | - | - | *OsFRDL1* |
| Os07g0673200 | -1.1781 | - | - | - | *OsNLA1* |
| Os06g0325200 | - | - | -1.4943 | - | *OsPT10* |
| Os02g0767500 | 1.3455 | - | - | - | *OsPT15* |
| Os03g0263400 | 1.1898 | - | - | - | *OsPT16* |
| Os09g0454600 | -1.0102 | - | - | - | *OsPT19* |
| Os01g0110100 | - | -1.4182 | - | - | *Pho1* |
| Os06g0493600 | - | - | 1.4977 | - | *Pho1* |
| Os04g0186400 | - | - | - | -1.9366 | *PT4* |
| Os10g0444700 | - | - | - | -1.7502 | *PT8* |
| Os03g0575200 | -1.4416 | - | - | - | *OsHAK16* |
| Os06g0270200 | - | -1.182 | - | - | *OsHAK24* |
| Os09g0448200 | - | - | -2.3351 | - | *OsHAK17* |
| Os07g0232800 | - | - | - | -2.4032 | *OsZIP8* |
| Os03g0195300 | - | -1.4855 | - | - | *OsSultr2* |
| Os03g0195450 | - | - | - | -1.1243 | *OsSultr2* |
| Os03g0161200 | - | - | -4.2905 | - | *OsSultr3* |
| Os01g0719300 | - | 2.085 | - | - | *OsSultr3* |
| Os06g0143700 | - | -2.4041 | - | - | *OsSultr3* |
| Os09g0240500 | -1.3592 | - | - | - | *OsSultr4* |
| Os01g0221600 | -1.8649 | - | - | - | *OsALMT4* |
| Os06g0331900 | - | - | 2.2388 | - | *OsALMT9* |
| Os07g0187900 | 1.0582 | - | - | - | Similar to nitrate and chloride transporter |
| Os01g0704100 | - | -2.0529 | - | - | *NRT2.3* |
| Os06g0706400 | - | -1.1286 | - | - | *OsPTR9* |
| Os02g0699000 | - | -1.353 | - | - | *OsNPF7.2* |
| Os03g0235900 | - | - | 1.0146 | - | *OsNRT1* |
| Os10g0111300 | - | - | 2.5237 | - | Similar to Nitrate transporter |
| Os02g0112100 | - | - | 1.8393 | - | *NRT2.1* |
| Os03g0800000 | - | - | - | 1.5811 | Similar to Nitrate and chloride transporter |
| Os10g0370700 | - | - | - | -1.286 | Similar to Nitrate transporter (Fragment) |
| Os02g0745100 | - | -1.693 | - | - | *LSI1* |

Treatments were denoted as: T1=As_10µM_, T2=As_10µM_+P_50µM_, T=3As_10µM_+*S.i*, T4=As+*S.i*+P_50µM_.

**Table S7.** Inclusively expressed phytohormone-related differential transcripts (log_2_ fold change) observed in response to As/*S. indica*/P interaction in ZZY-1 and GD-6.

| Gene ID | G1T1 *vs.* G2T1 | G1T2 *vs.* G2T2 | G1T3 *vs.* G2T3 | G1T4 *vs.* G2T4 | Description |
| --- | --- | --- | --- | --- | --- |
| Os11g0673200 | 1.1193 | - | - | - | Similar to Auxin-induced beta-glucosidase |
| Os03g0836800 | 8.543 | - | - | - | Similar to IAA-amino acid hydrolase 1 (EC 3.5.1.-) |
| Os04g0671900 | 1.0358 | - | - | - | *ARF8* |
| Os03g0175800 | 2.076 | - | - | - | *BG1* |
| Os10g0479900 | 1.0914 | - | - | - | *OsARF22* |
| Os02g0628600 | 1.2027 | - | - | - | *OsARF8* |
| Os03g0780350 | -2.3154 | - | - | - | Similar to SAUR1 - auxin-responsive SAUR family member |
| Os06g0597000 | 1.4348 | - | - | - | *OsIAA23* |
| Os09g0491740 | -1.5714 | - | - | - | *OsPILS1* |
| Os03g0660300 | -2.6796 | - | - | - | *OsSAUR15* |
| Os01g0645400 | 2.5443 | - | - | - | *YUCCA1* |
| Os03g0342900 | - | -1.1602 | - | - | Dormancy auxin associated family protein |
| Os02g0164900 | - | -1.0365 | - | - | *OsARF6a* |
| Os05g0143800 | - | -1.2094 | - | - | *OsGH3-6* |
| Os05g0186900 | - | -1.3635 | - | - | *OsIAA16* |
| Os02g0643800 | - | -1.1024 | - | - | *OsSAUR11* |
| Os09g0545400 | - | -1.0581 | - | - | *OsSAUR40* |
| Os03g0347700 | - | 1.3156 | - | - | *WIN1* |
| Os03g0323500 | - | - | 1.0914 | - | Arf GTPase activating protein family protein |
| Os01g0785400 | - | - | 2.9672 | - | *OsGH3-1* |
| Os06g0166500 | - | - | -1.5631 | - | *OsIAA20* |
| Os02g0805100 | - | - | 1.8283 | - | *OsIAA9* |
| Os09g0554300 | - | - | 1.4273 | - | *OsPILS7a* |
| Os01g0802700 | - | - | -1.3646 | - | *OsPIN9* |
| Os03g0660400 | - | - | -2.2917 | - | *OsSAUR16* |
| Os04g0662200 | - | - | 2.025 | - | *OsSAUR22* |
| Os09g0437400 | - | - | 1.7901 | - | *OsSAUR38* |
| Os09g0546700 | - | - | 4.2528 | - | *OsSAUR51* |
| Os08g0529000 | - | - | -1.6541 | - | *PIN5B* |
| Os09g0547033 | - | - | - | 4.6632 | Similar to SAUR44 - auxin-responsive SAUR family member |
| Os09g0555100 | - | - | - | -1.3981 | *OsPILS7b* |
| Os06g0702000 | - | - | - | -1.0763 | *OsSAUR28* |
| Os06g0714300 | - | - | - | -1.3053 | *OsSAUR29* |
| Os07g0227600 | -1.0754 | - | - | - | *OsERF* |
| Os05g0473300 | -1.0762 | - | - | - | *OsERF* |
| Os10g0523900 | -2.6076 | - | - | - | *OsERF* |
| Os03g0313100 | -2.1369 | - | - | - | *AP2/EREBP* |
| Os04g0398000 | -1.115 | 2.6178 | - | - | *OsERF101* |
| Os06g0181700 | - | -1.0426 | - | - | *OsWR4* |
| Os01g0720400 | - | -1.2826 | - | - | *OsACP1* |
| Os01g0693400 | - | -1.4514 | - | - | *AP2/EREBP* |
| Os05g0549800 | - | 1.7953 | - | - | *AP2/EREBP* |
| Os06g0639200 | - | -1.4204 | - | - | *AP2/EREBP* |
| Os04g0529100 | - | 1.3399 | - | - | *OsERF* |
| Os07g0410300 | - | -1.2597 | - | - | *OsERF* |
| Os03g0183300 | - | 2.4865 | - | - | *OsERF* |
| Os11g0242300 | - | -2.0015 | - | - | *OsERF* |
| Os02g0676800 | - | -2.9519 | - | - | *OsERF* |
| Os04g0547600 | - | 3.0622 | - | - | *OsERF* |
| Os04g0399800 | - | 3.9663 | - | - | *OsERF* |
| Os02g0520000 | - | - | - | - | *OsERF* |
| Os01g0868000 | - | - | -1.2484 | - | *ERF99* |
| Os05g0437100 | - | - | -1.0847 | - | Pathogenesis-related TF and ERF domain containing protein |
| Os08g0442400 | - | - | -2.1668 | - | *AP2/EREBP* |
| Os11g0129700 | - | - | 1.1038 | - | *AP2/EREBP* |
| Os12g0168100 | - | - | -1.021 | - | *DERF8* |
| Os11g0168500 | - | - | 1.1788 | - | *OsERF* |
| Os05g0448700 | - | - | 1.0205 | - | *OsERF* |
| Os08g0173700 | - | - | 3.5229 | - | *OsERF* |
| Os10g0371100 | - | - | -1.182 | - | *OsERF* |
| Os03g0750000 | - | - | -1.3434 | - | Similar to ethylene-responsive protein |
| Os03g0818800 | - | - | - | 1.0019 | *AP2/EREBP* |
| Os05g0420300 | - | - | - | -3.6931 | *ERF131* |
| Os08g0521600 | - | - | - | -4.3953 | *OsERF* |
| Os12g0478200 | -1.1231 | - | - | - | *OsABA45* |
| Os07g0164900 | -1.0182 | - | - | - | *OsAAO* |
| Os02g0703600 | - | -1.5042 | - | - | *OsABA8OX1* |
| Os02g0734500 | - | - | -4.0151 | - | Similar to ABA-induced protein |
| Os11g0575600 | - | -3.3063 | - | - | *OsLOX10* |
| Os05g0586200 | -1.5372 | - | - | - | *JAR1* |
| Os03g0396500 | - | -4.8332 | - | - | *OsJAZ* |
| Os02g0218800 | - | - | - | -1.9152 | *AOS4* |
| Os09g0439200 | - | 1.0455 | - | - | *TIFY10C* |
| Os06g0313320 | - | -4.6876 | - | - | Similar to Jasmonate O-methyltransferase |
| Os02g0218700 | - | - | -1.463 | - | *HPL2* |
| Os01g0900400 | - | - | -3.099 | - | *EG1* |
| Os03g0328000 | 1.19 | - | - | - | Dedicator of cytokinesis family protein |
| Os02g0557800 | 1.3168 | - | - | - | *OSRR2* |
| Os08g0460600 | - | -1.2371 | - | - | *OsCKX11* |
| Os07g0546000 | 1.721 | - | - | - | *IPI* |
| Os03g0810100 | - | 1.3766 | - | - | *OsIPT4* |
| Os04g0319800 | - | - | 3.3633 | - | Cytokinin-O-glucosyltransferase 2 (Zeatin O- glucosyltransferase 2) |
| Os01g0940000 | - | - | -1.393 | - | *CKX4* |
| Os02g0796500 | - | - | -1.1166 | - | *ORR3* |
| Os07g0241700 | - | - | - | -1.197 | Similar to Cytokinin-N-glucosyltransferase 1 |
| Os04g0371000 | - | - | - | -1.3053 | Four-helical cytokine family protein |
| Os02g0220000 | - | - | - | 1.0832 | *OsCKX6* |
| Os07g0580500 | 1.2429 | - | - | - | *BZR1* |
| Os05g0207500 | 1.3857 | - | - | - | *GSK2* |
| Os05g0324600 | - | 2.1963 | - | - | *OsOFP19* |
| Os06g0714600 | - | -1.6521 | - | - | *OsPRA2* |
| Os06g0274300 | - | 1.9637 | - | - | *SEPK1* |
| Os09g0459200 | - | 1.1667 | - | - | *SG1* |
| Os03g0227700 | - | -1.9985 | - | - | *OsDWF4* |
| Os02g0156300 | - | - | -1.1026 | - | Similar to Ser/threo-protein kinase BRI1-like 2 precursors (EC 2.7.1.37) |
| Os05g0414700 | - | - | - | -1.0506 | Similar to BRI1-associated receptor kinase 1 |
| Os02g0683500 | - | - | - | -2.3599 | *RAV6* |
| Os02g0103700 | 1.5721 | - | - | - | Similar to 60S ribosomal protein L9 (Gibberellin-regulated protein GA) |
| Os09g0485900 | 1.5224 | - | - | - | Similar to 60S ribosomal protein L9 (Gibberellin-regulated protein GA) |
| Os08g0474866 | - | -1.8787 | - | - | Similar to gibberellin receptor GID1L2 |
| Os01g0571100 | - | 2.3934 | - | - | Similar to gibberellin responsive1 |
| Os07g0583600 | - | -1.185 | - | - | CIGR2, Chitin-inducible gibberellin-responsive protein |
| Os05g0432200 | - | - | -1.3482 | - | Similar to Gibberellin-regulated protein 2 precursor |
| Os03g0607200 | - | - | - | -1.2632 | Gibberellin regulated protein family protein |
| Os10g0115550 | - | - | - | 4.6393 | Similar to gibberellin-regulated family protein |
| Os11g0669100 | - | - | - | 1.0291 | Calmodulin binding protein-like family protein |
| Os03g0778000 | - | - | - | 1.0889 | Calmodulin binding protein-like family protein |

Treatments were denoted as: T1=As_10µM_, T2=As_10µM_+P_50µM_, T=3As_10µM_+*S.i*, T4=As+*S.i*+P_50µM_.
