# Supplementary material for: Pan-transcriptomic Profiling Demarcates Serendipita Indica-Phosphorus Mediated Tolerance Mechanisms in Rice Exposed to Arsenic Toxicity
Source: Rice (N Y). 2023 Jun 24;16:28. doi: 10.1186/s12284-023-00645-0 (PMC10290630; doi:10.1186/s12284-023-00645-0)
Supplement: Supplementary file 2 — Additional file 2:Fig. S1. Validation of data obtained from RNA-Seq with real-time quantitative reverse transcription PCR. Fig. S2. Summary of gene mapping ratio as observed across the samples including all treatments between ZZY-1 (G1) and GD-6 (G2). Fig. S3. Venn diagram representing exclusive and inclusive DEGs expressed for G1 = ZZY1 (A), and G2 = GD-6 (B), in response to As alone, As + P, As + S.i and As + S.i + P treatments along with control. Fig. S4. Principal component analysis (PCA) of As, P and S. indica-induced transcripts across the treatments in two rice genotypes. Fig. S5. Time series analysis illustrating Mfuzz results. T1 = As10µM, T2 = As10µM + P50µM, T3 = As10µM + S.i, T4 = As10µM + S.i + P50µM and T5 = control. Fig. S6. Distribution of differentially expressed transcripts using color module (A), and cluster dendrogram (B). Fig. S7. KEGG analysis of DEGs presented as scatter plot showing 20 most significantly enriched pathways for: (A) G1T1 vs. G2T1, (B) G1T2 vs. G2T2, and (C) G1T3 vs. G2T3 comparisons. Fig. S8. Relative comparative gene ontology (GO) in ZZY-1 under As10 (A), As10µM + P50µM (B), and As10µM + S.i (C) in comparison to GD-6. Fig. S9. Heat map presenting the expression profile of mutually expressed stress responsive DEGs. Fig. S10. Heat map showing the expression profile of inclusively expressed heavy metal associated transporters. Fig. S11. Heat map representing the expression profile of inclusively expressed ABC transporters. Fig. S12. Hierarchical heat map depicting the expression profile of inclusively expressed genes related to auxin biosynthesis and signaling. Fig. S13. Hierarchical heat map illustrating the expression profile of inclusively expressed genes related to the biosynthesis and signaling of ethylene. Fig. S14. Hierarchical heatmap representation of differentially expressed GELP transcripts (inclusively/exclusively) observed in response to As alone, As + P, As + S.i and As + S.i + P along with control, in two rice geno [file 12284_2023_645_MOESM2_ESM.docx]

**Pan-transcriptomic profiling demarcates *Serendipita indica*-phosphorus mediated tolerance mechanisms in rice exposed to arsenic toxicity**

Shafaque Sehar ^1†^, Muhammad Faheem Adil^1†^, Syed Muhammad Hassan Askri^1^, Qidong Feng^1^, Dongming Wei^1^, Falak Sehar Sahito^2^, Imran Haider Shamsi^1*^

^1^Zhejiang Key Laboratory of Crop Germplasm Resource, Department of Agronomy, College of Agriculture and Biotechnology, Zhejiang University, Hangzhou 310058, China

^2^ Dow International Medical College, Dow University of Health Sciences, Karachi 74200, Pakistan

*Corresponding author’s email: [drimran@zju.edu.cn](mailto:drimran@zju.edu.cn)

^†^These authors contributed equally to this study


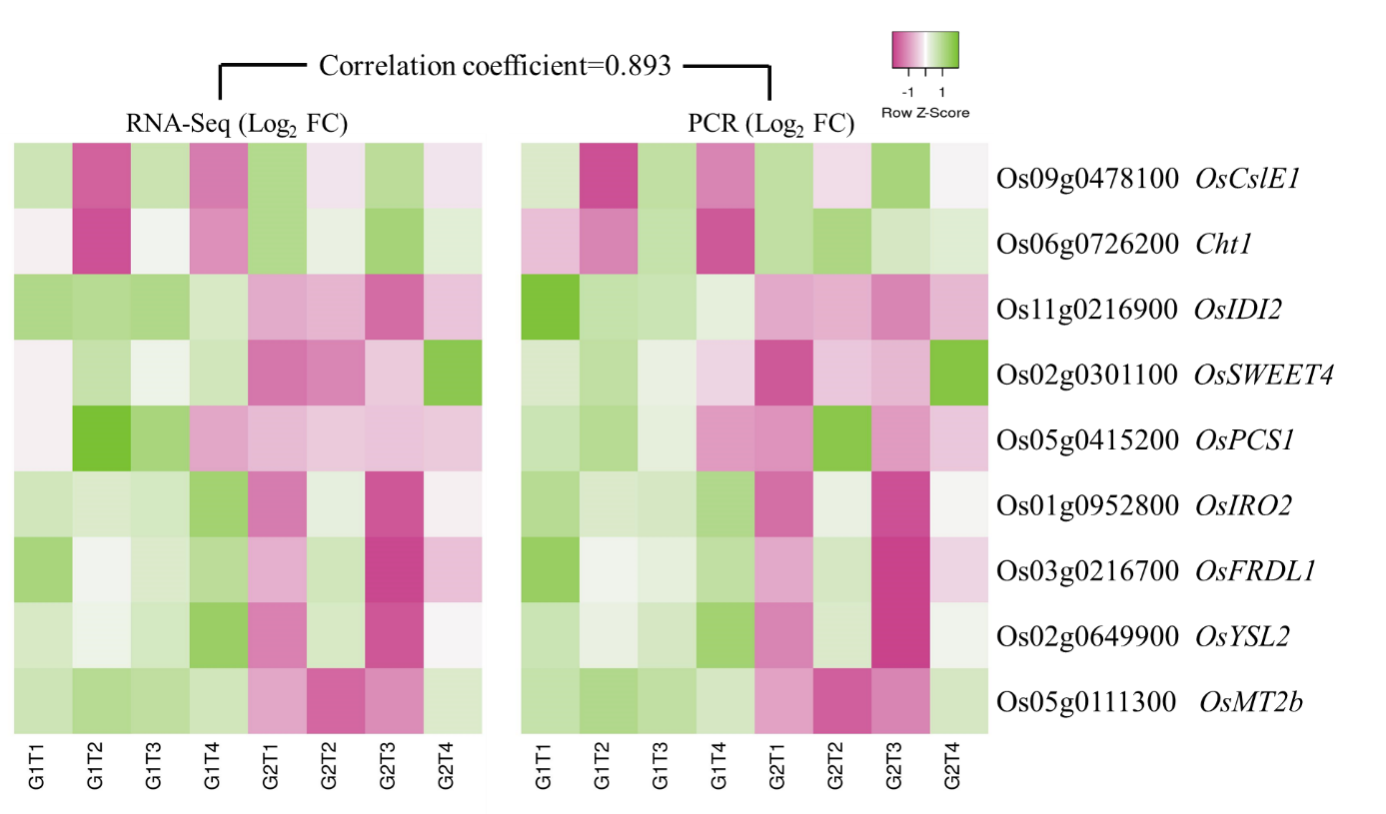


**Fig. S1.** Validation of data obtained from RNA-Seq with real-time quantitative reverse transcription PCR.


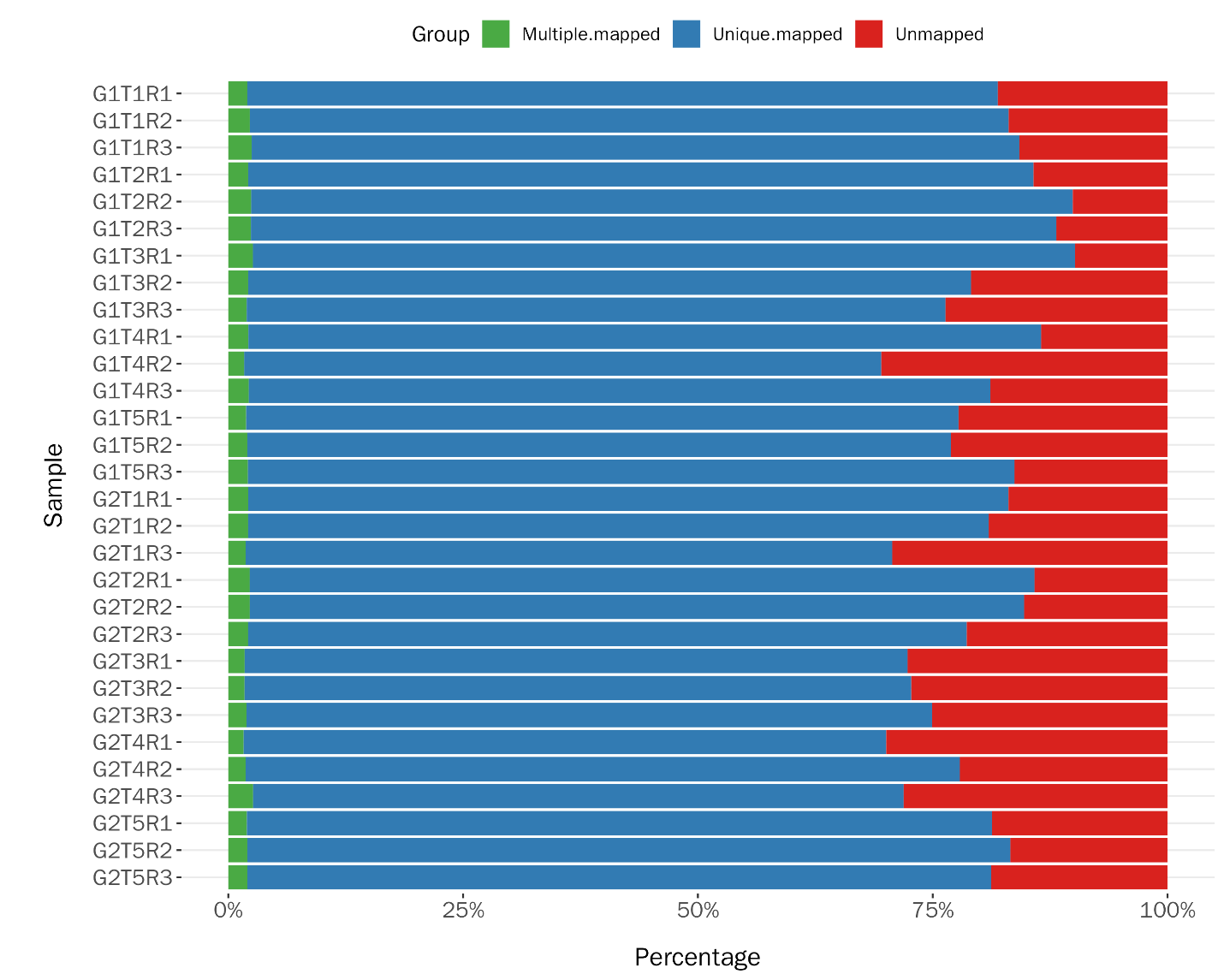


**Fig. S2.** Summary of gene mapping ratio as observed across the samples including all treatments between ZZY-1 (G1) and GD-6 (G2).


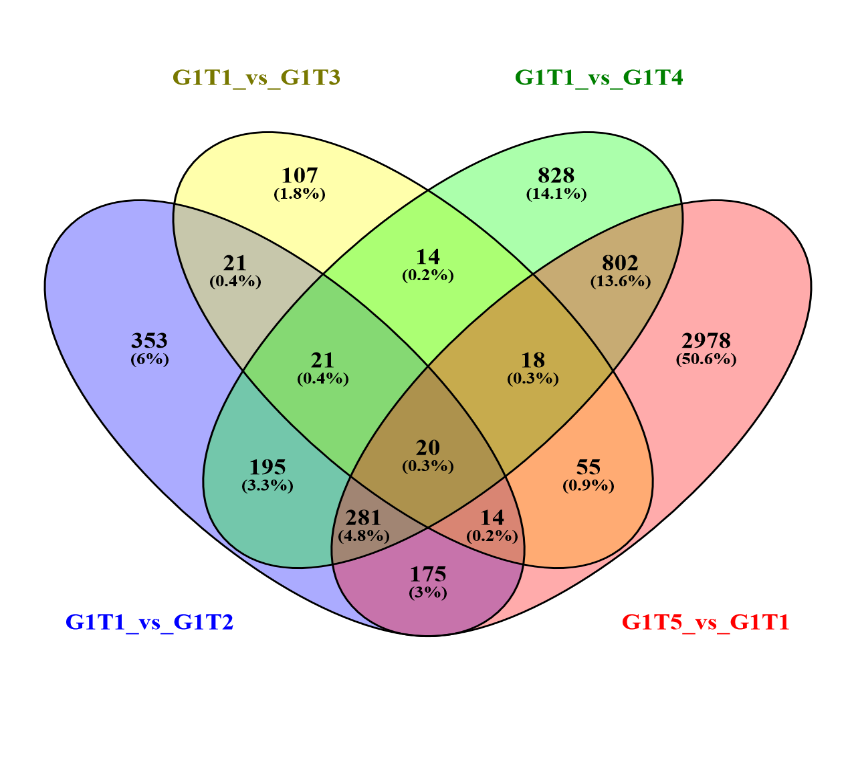

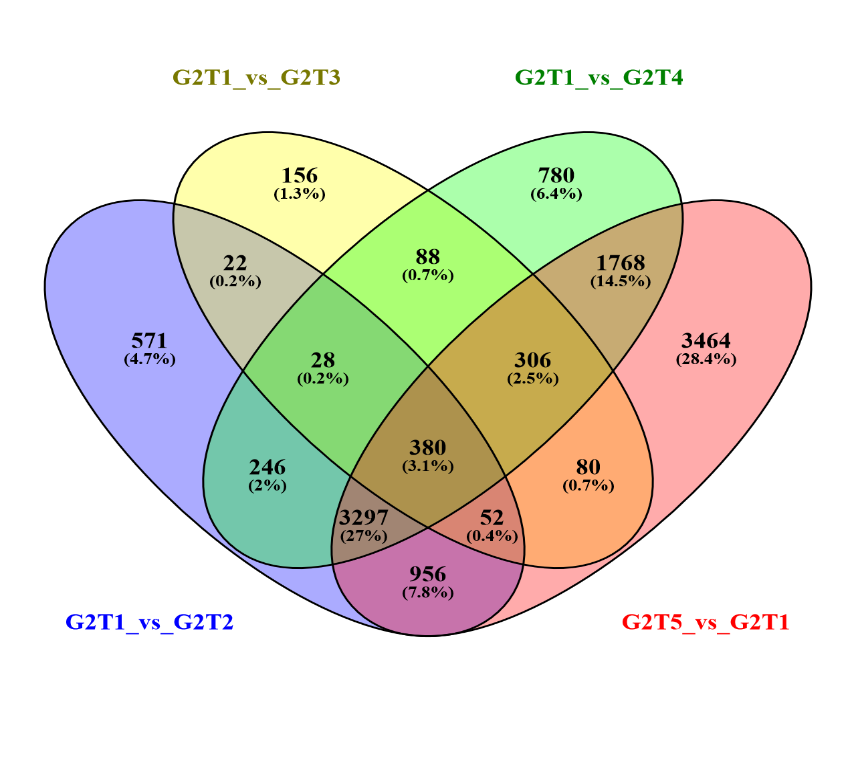


(A)

(B)

**Fig. S3.** Venn diagram representing exclusive and inclusive DEGs expressed for G1=ZZY1 (A), and G2=GD-6 (B), in response to As alone (T1), As+P (T2), As+*S.i* (T3) and As+*S.i*+P (T4) treatments along with control (T5).


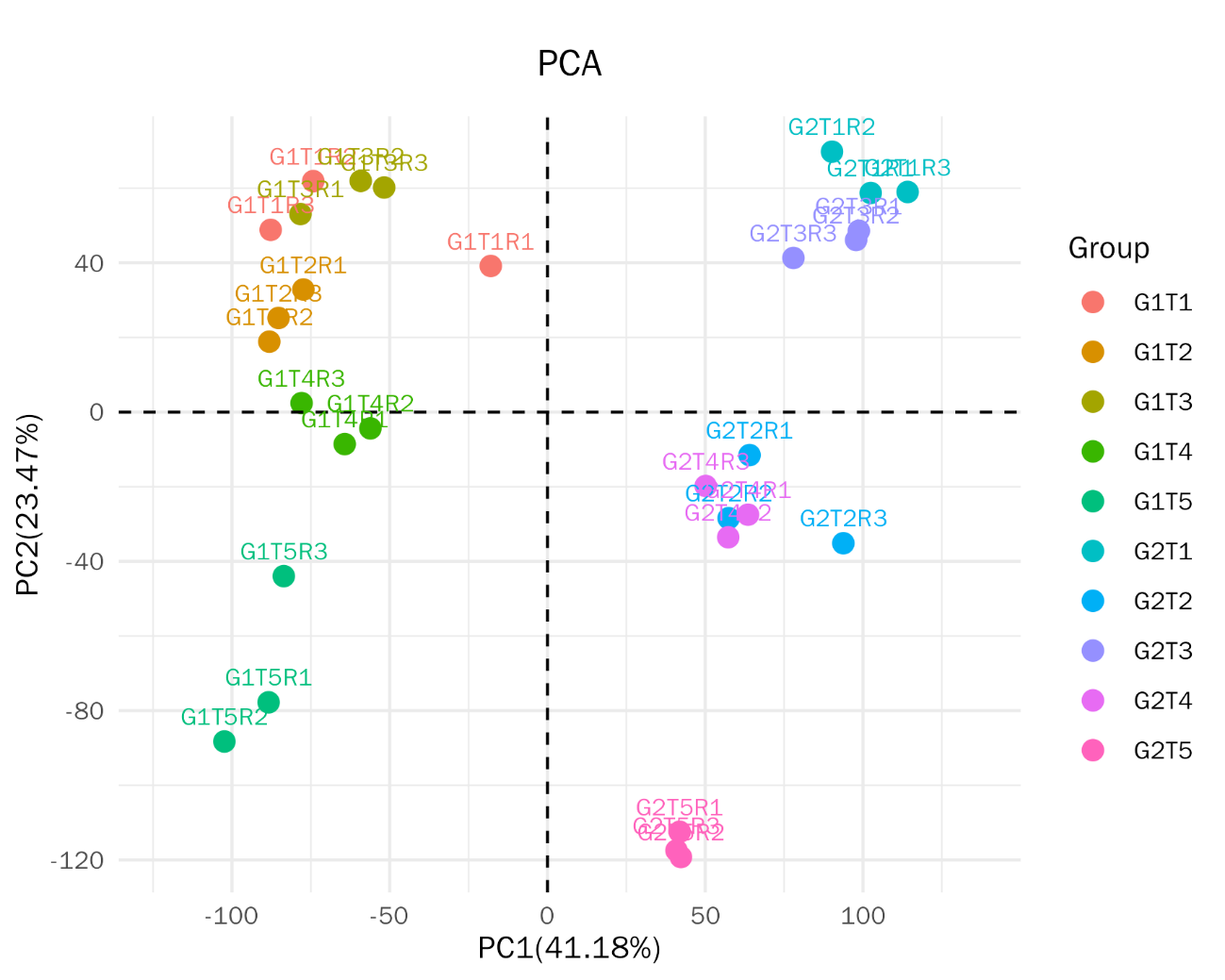


**Fig S4.** Principal component analysis (PCA) of As, P and *S. indica*-induced transcripts across the treatments in two rice genotypes (G1—ZZY-1 and G2—GD-6). T1= As_10µM_, T2=As_10µM_+P_50µM_, T3=As_10µM_+*S.i*, T4=As_10µM_+*S.i*+P_50 µM_, and T5=control.


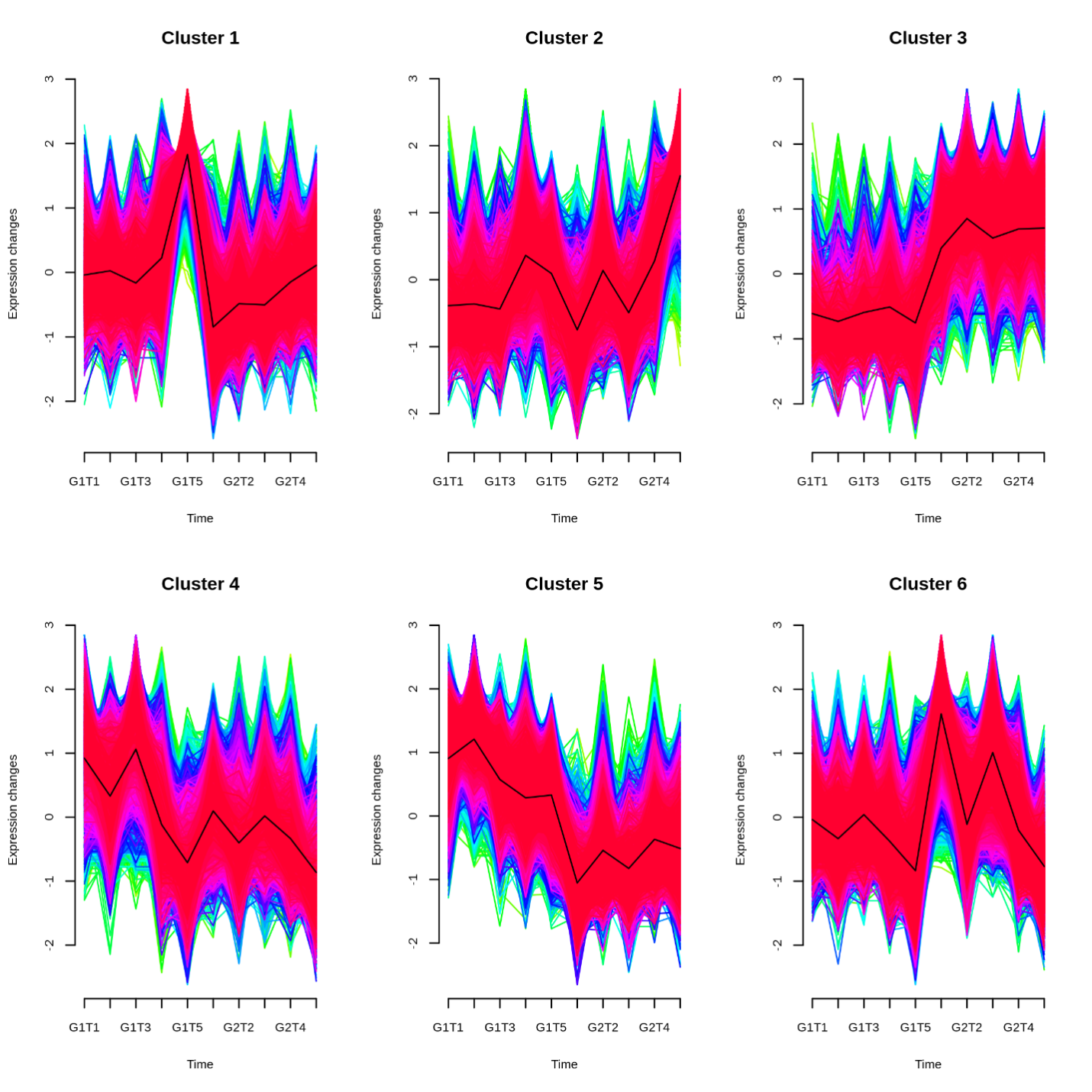


**Fig. S5**. Time series analysis illustrating Mfuzz results. T1= As_10_, T2=As_10_+P_50_, T3=As_10_+*S.i*, T4=As_10_+*S.i*+P_50_ and T5=control; G1=ZZY-1 and G2=GD-6.


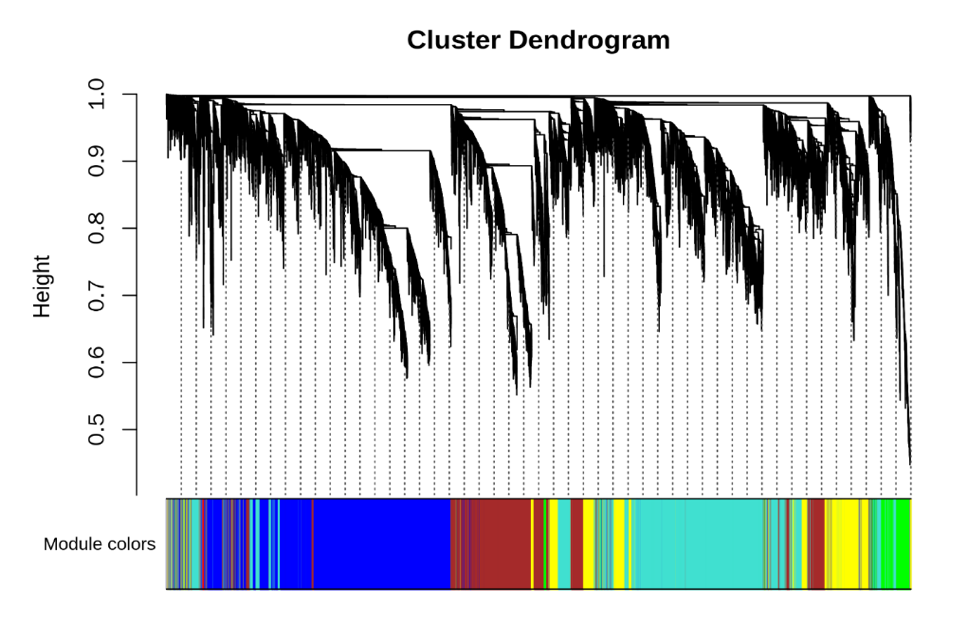

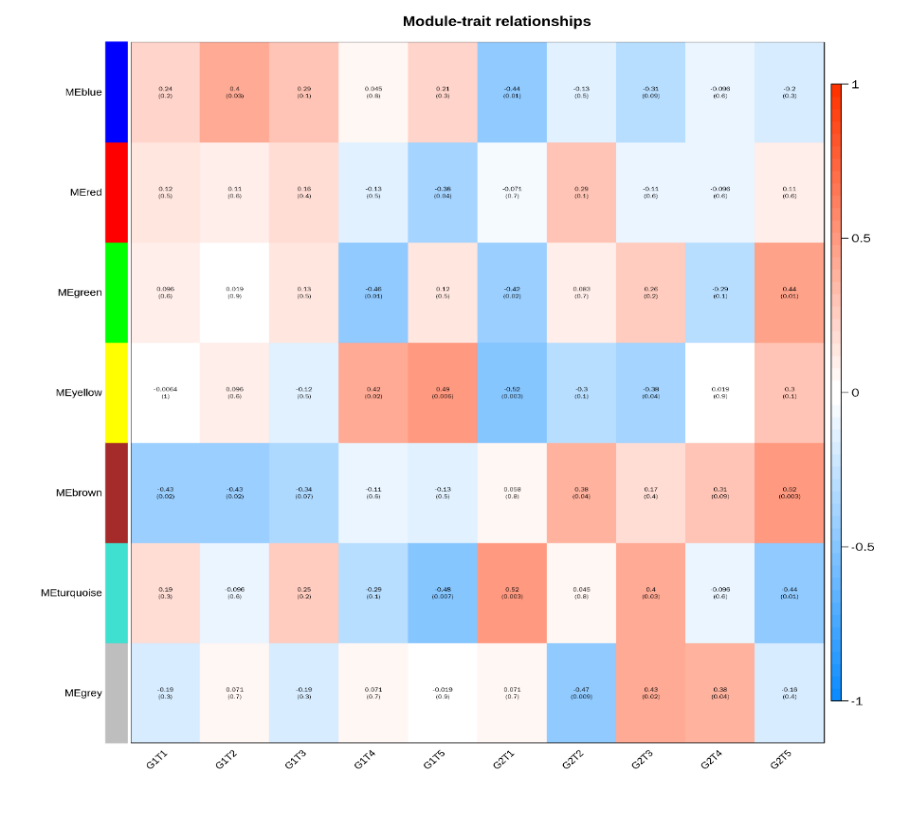


(A)

(B)

**Fig. S6.** Distribution of differentially expressed transcripts using color module (A), and cluster dendrogram (B).


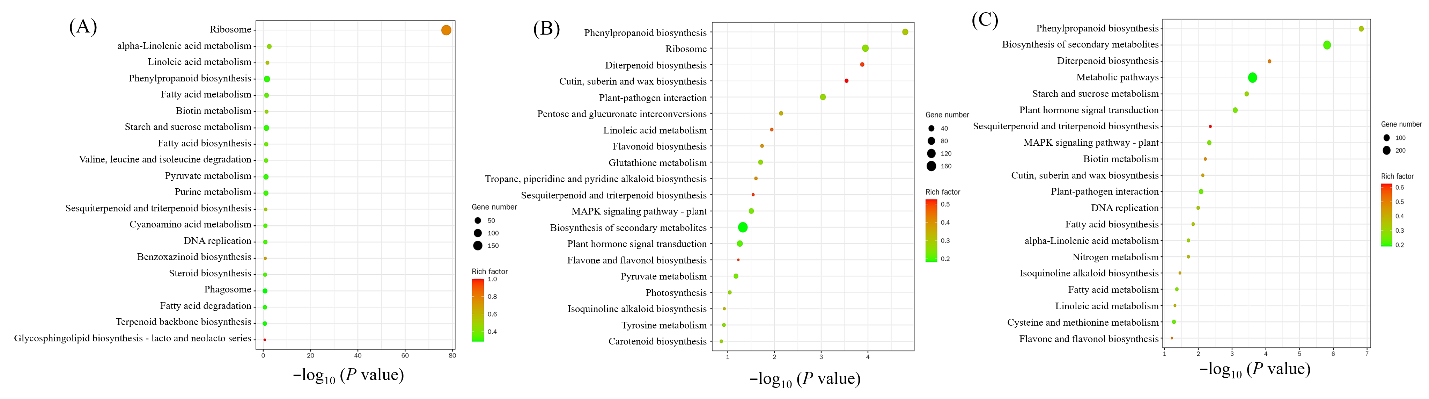


**Fig. S7.** KEGG analysis of DEGs presented as scatter plot showing 20 most significantly enriched pathways for: (A) G1T1 *vs.* G2T1, (B) G1T2 *vs.* G2T2, and (C) G1T3 *vs.* G2T3 comparisons. Each circle represents a pathway and the size of the circle represents the number of genes enriched in the pathway. The ordinate represents the pathway name and the abscissa represents the rich factor, which compares the ratio of genes annotated to a pathway among the DEGs to the ratio of genes annotated to that pathway among all genes. The color of the circle represents the *q* value, where smaller values equate to more reliable enrichment significances of the DEGs in the pathway


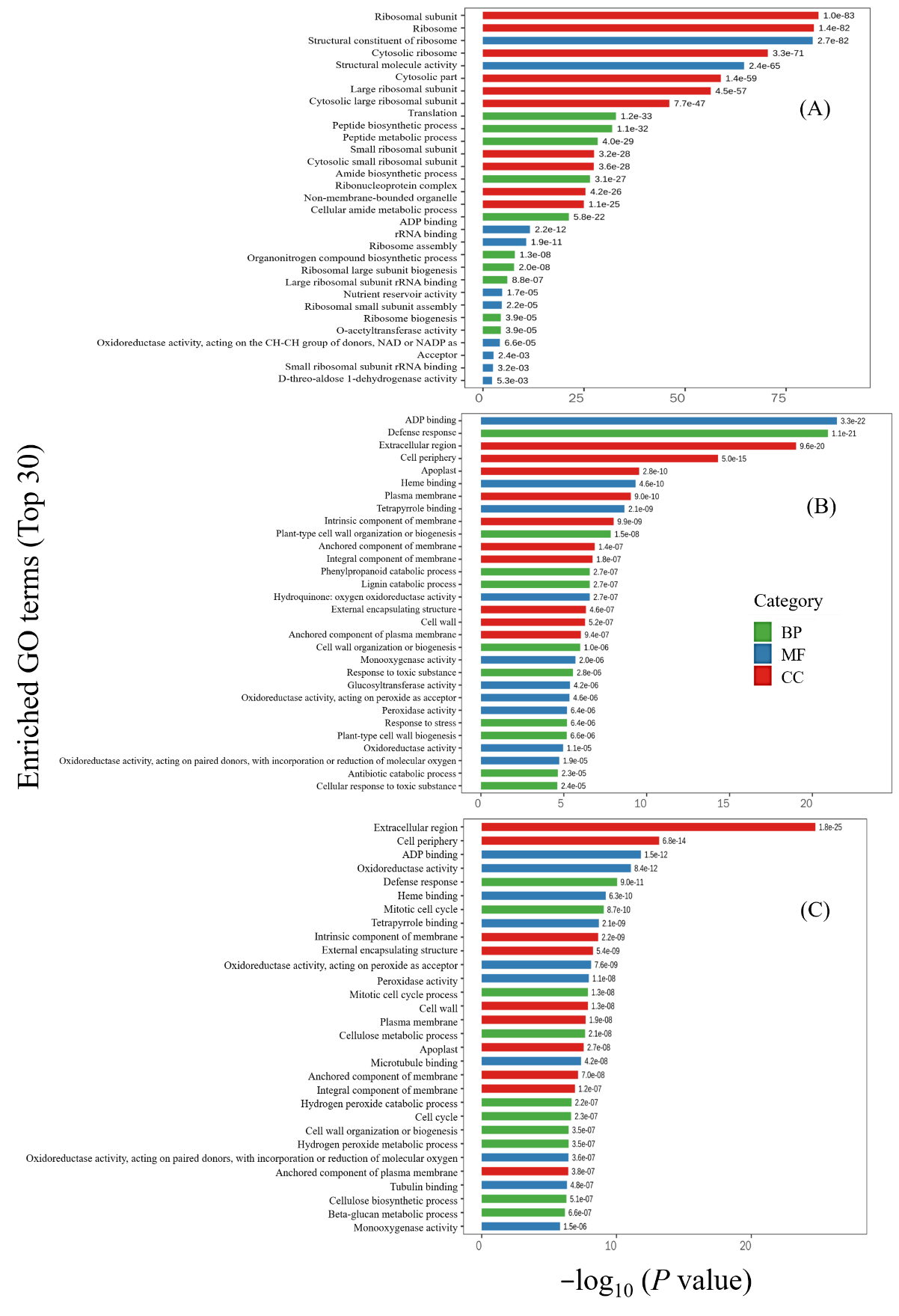


**Fig. S8.** Relative comparative gene ontology (GO) in ZZY-1 under As_10_ (A), As_10_+P_50_ (B), and As_10_+*S.i* (C) in comparison to GD-6. GO terms were sorted based on *q*-values (< 0.05) and belonged to biological processes, cellular components, and molecular functions were shown in green, red and blue color, respectively.


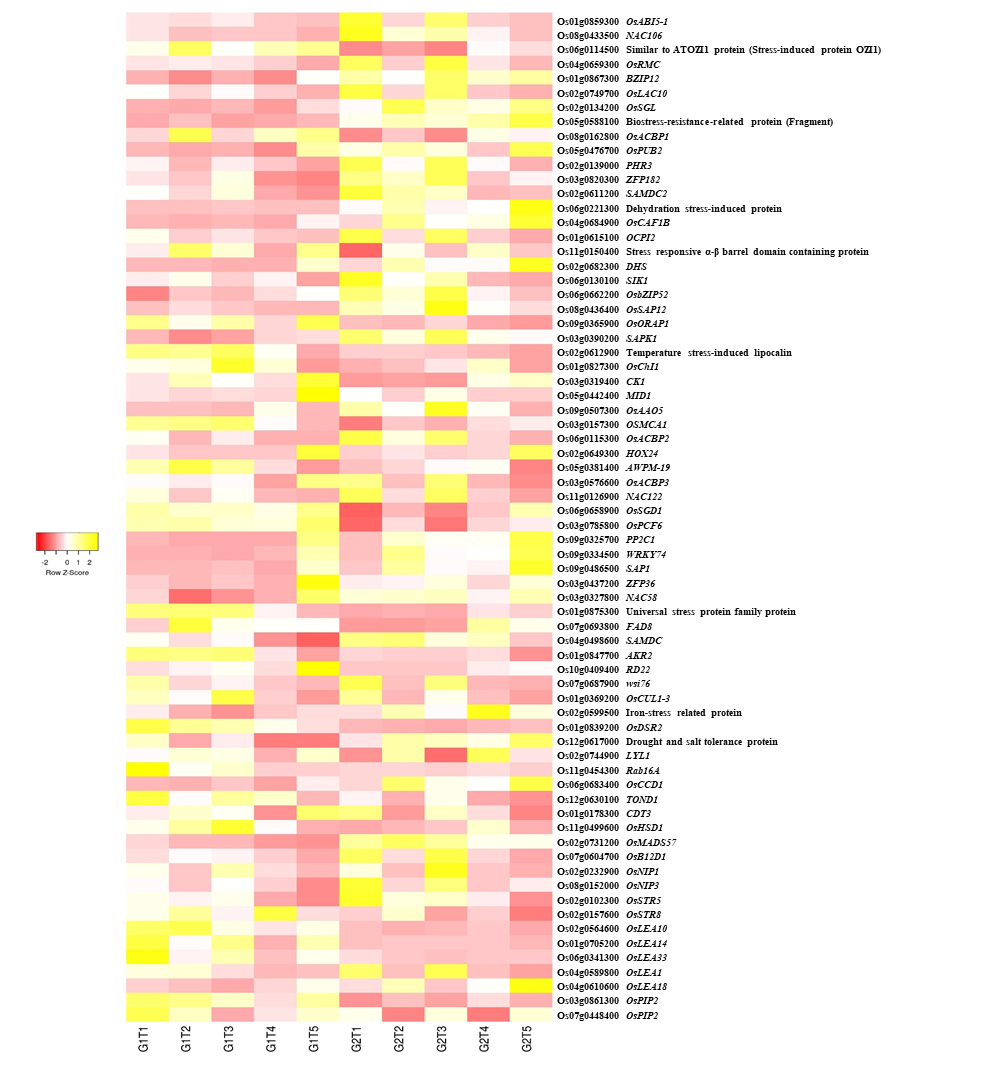


**Fig. S9**. Heat map presenting the expression profile of mutually expressed stress responsive DEGs in response to As alone (T1), As+P (T2), As+*S.i* (T3) and As+*S.i*+P (T4) treatments along with control (T5), in the two rice genotypes ZZY-1 (G1) and GD-6 (G2).


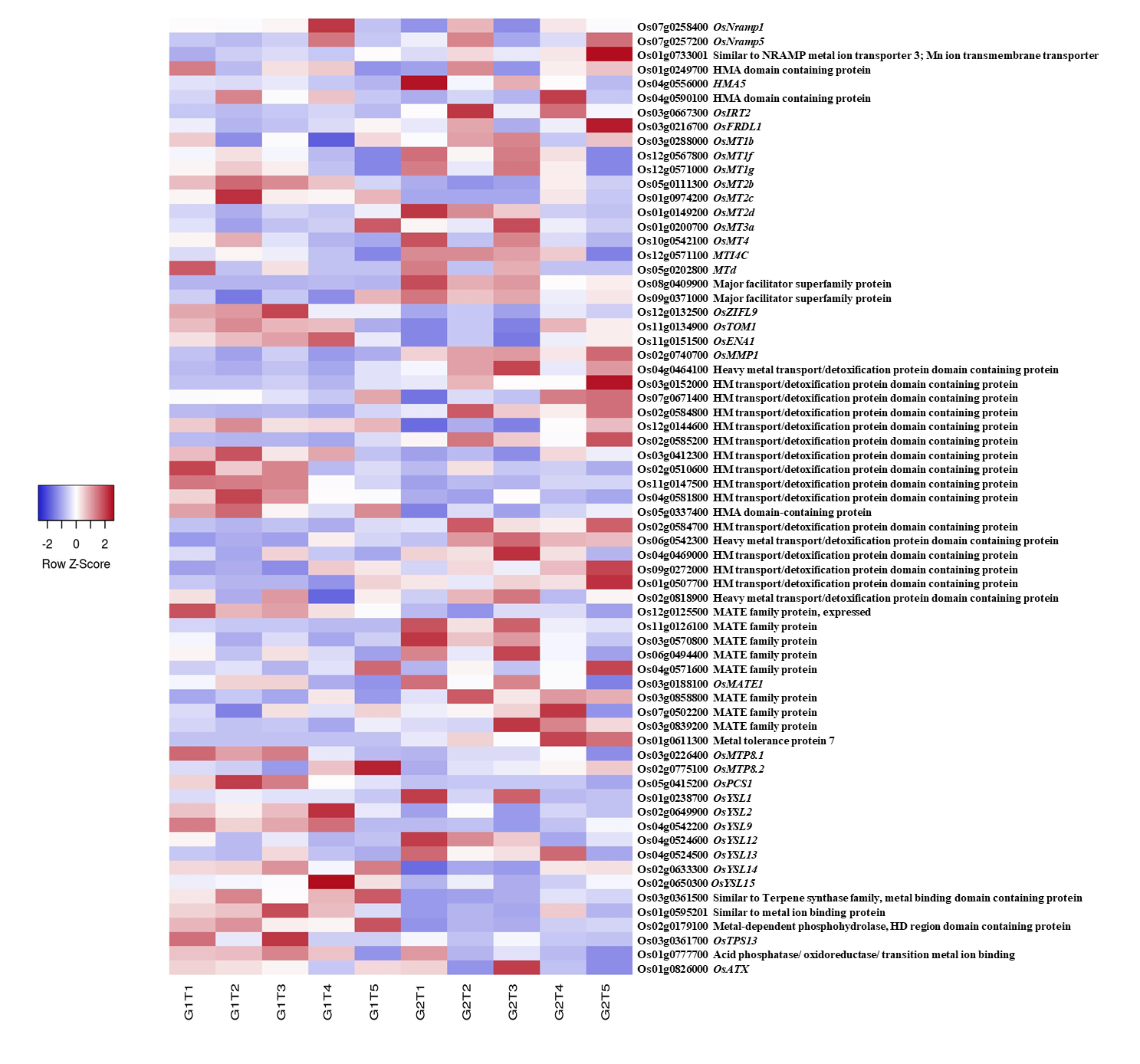


**Fig. S10**. Heat map showing the expression profile of inclusively expressed heavy metal associated transporters in response to As alone (T1), As+P (T2), As+*S.i* (T3) and As+*S.i*+P (T4) treatments along with control (T5), in the two rice genotypes ZZY-1 (G1) and GD-6 (G2).


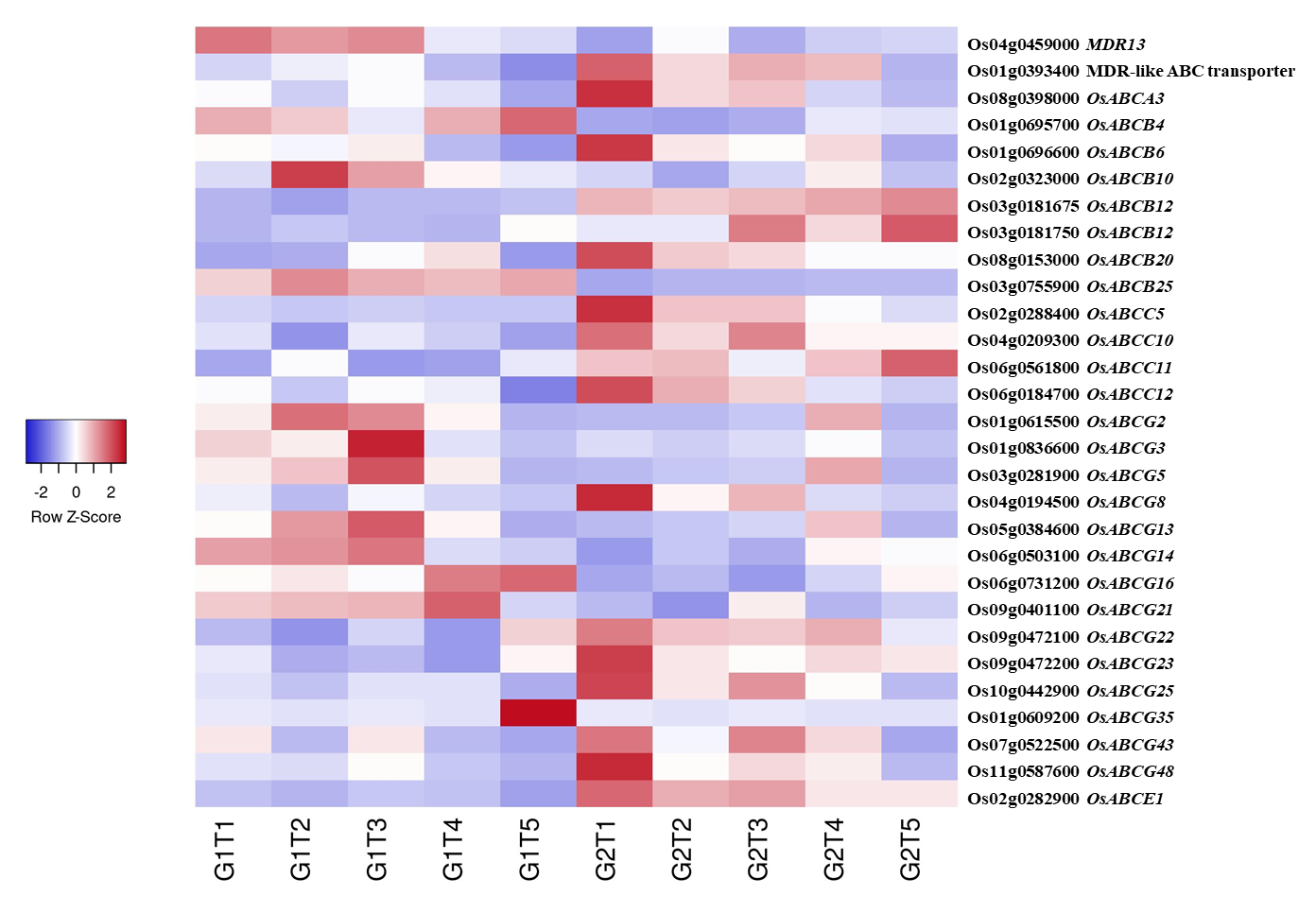


**Fig. S11**. Heat map representing the expression profile of inclusively expressed ABC transporters in response to As alone (T1), As+P (T2), As+*S.i* (T3) and As+*S.i*+P (T4) treatments along with control (T5), in the two rice genotypes ZZY-1 (G1) and GD-6 (G2).


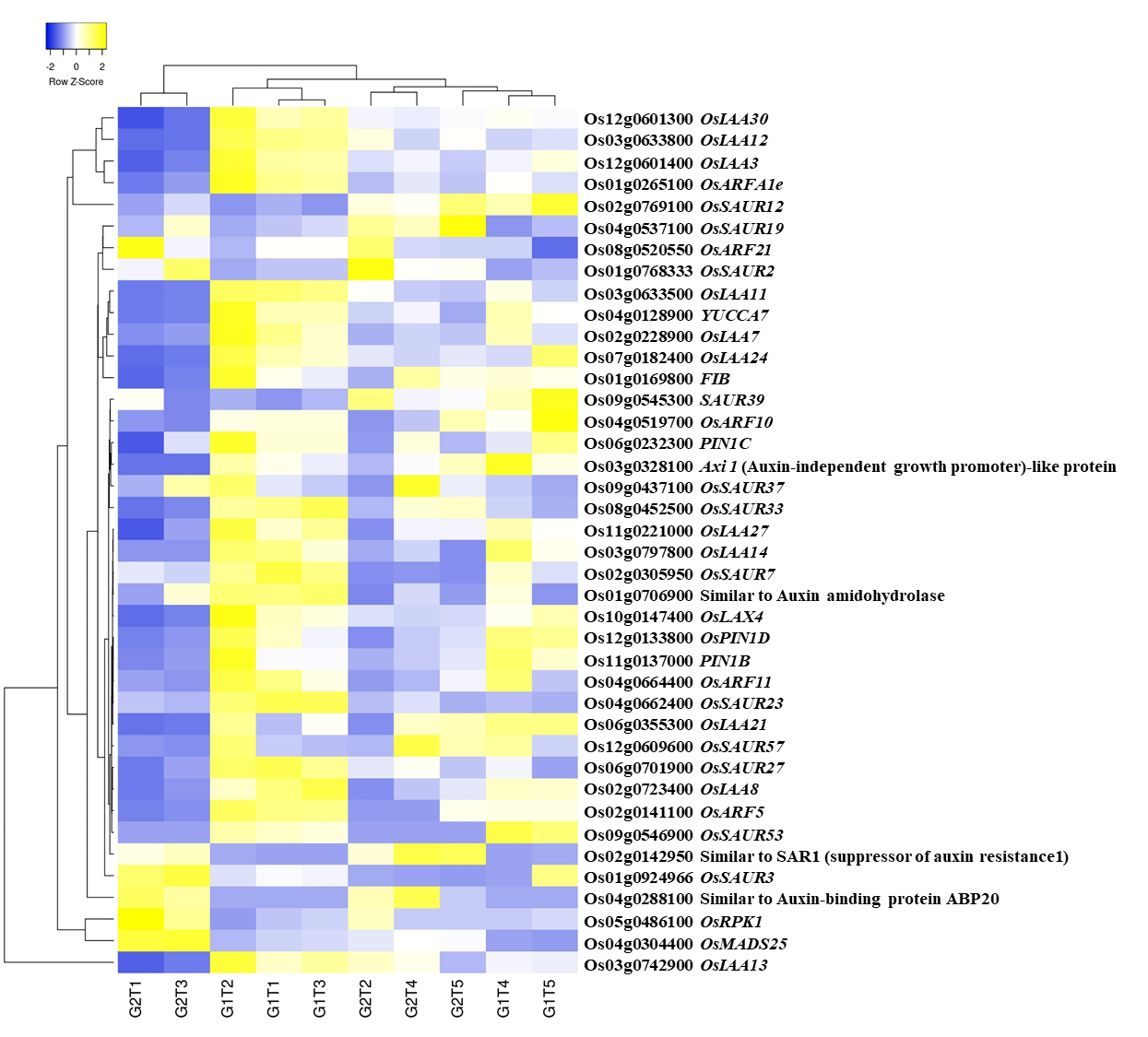


**Fig. S12.** Hierarchical heat map depicting the expression profile of inclusively expressed genes related to auxin biosynthesis and signaling in response to As alone (T1), As+P (T2), As+*S.i* (T3) and As+*S.i*+P (T4) treatments along with control (T5), in the two rice genotypes ZZY-1 (G1) and GD-6 (G2).


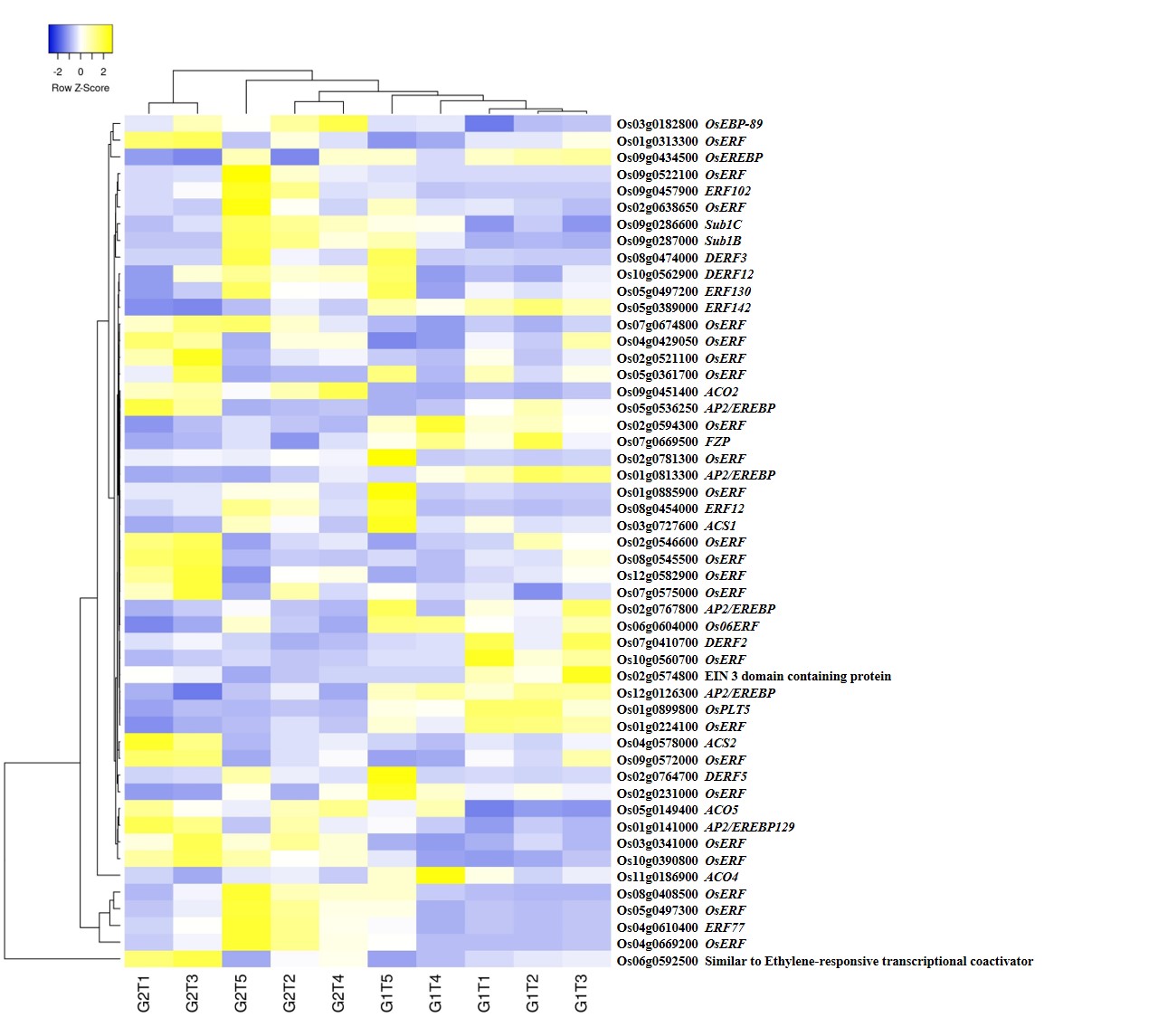


**Fig. S13.** Hierarchical heat map illustrating the expression profile of inclusively expressed genes related to the biosynthesis and signaling of ethylene in response to As alone (T1), As+P (T2), As+*S.i* (T3) and As+*S.i*+P (T4) treatments along with control (T5), in the two rice genotypes ZZY-1 (G1) and GD-6 (G2).


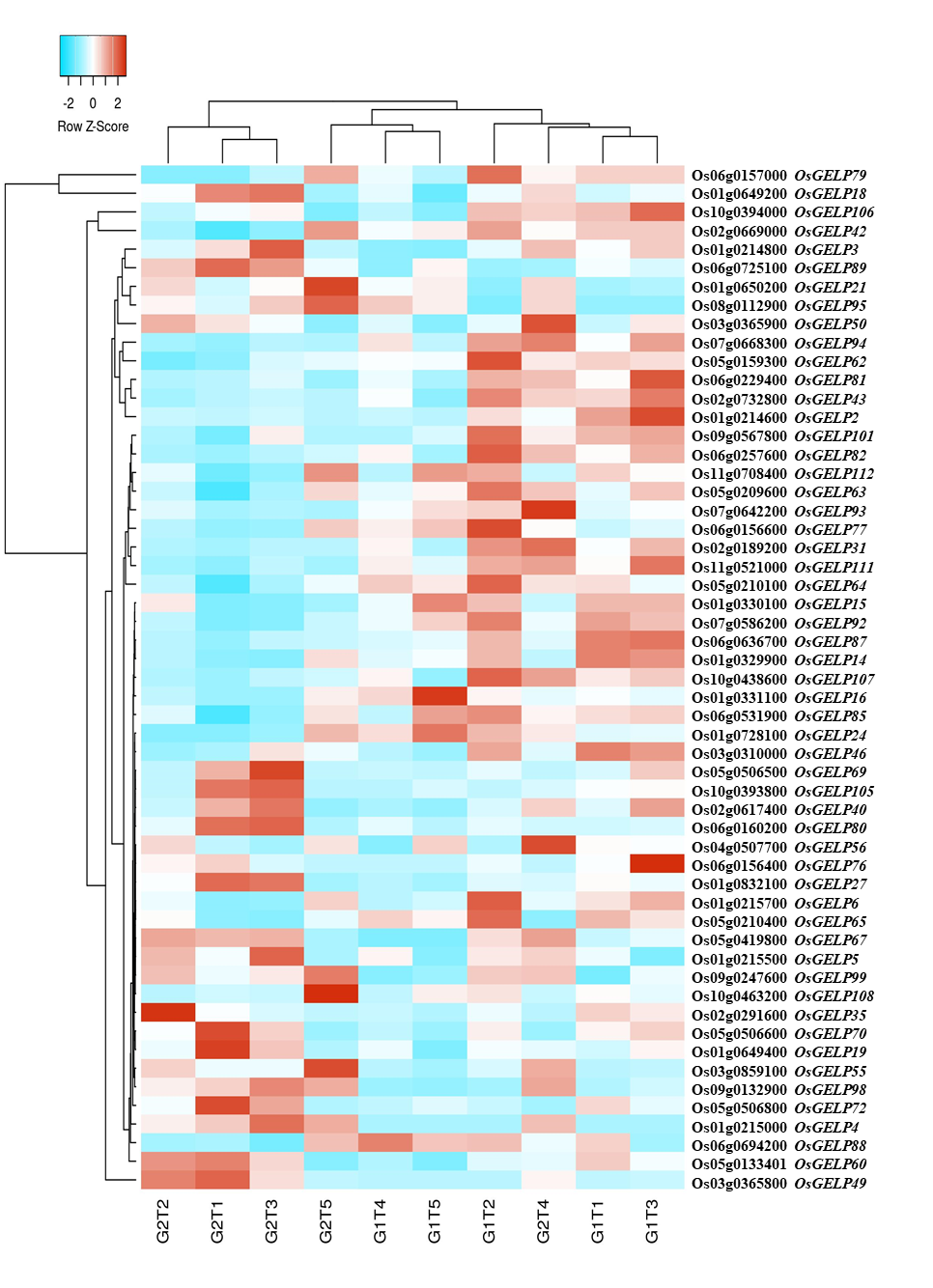


**Fig. S14.** Hierarchical heatmap representation of differentially expressed *GELP* transcripts (inclusively/exclusively) observed in response to As alone (T1), As+P (T2), As+*S.i* (T3), and As+*S.i*+P (T4) along with control (T5), in the two rice genotypes ZZY-1 (G1) and GD-6 (G2).
